# Bola‐Amphiphilic Glycodendrimers: New Carbohydrate‐Mimicking Scaffolds to Target Carbohydrate‐Binding Proteins

**DOI:** 10.1002/chem.202201400

**Published:** 2022-08-26

**Authors:** Wenzheng Zhang, Dinesh Dhumal, Xiaolei Zhu, Brigino Ralahy, Aleksandra Ellert‐Miklaszewska, Jing Wu, Erik Laurini, Yi‐Wen Yao, Chai‐Lin Kao, Juan L. Iovanna, Sabrina Pricl, Bozena Kaminska, Yi Xia, Ling Peng

**Affiliations:** ^1^ Chongqing Key Laboratory of Natural Product Synthesis and Drug Research Innovative Drug Research Center School of Pharmaceutical Sciences Chongqing University Chongqing 401331 P. R. China; ^2^ Centre Interdisciplinaire de Nanoscience de Marseille (CINaM) UMR 7325, Equipe Labellisé par La Ligue Aix-Marseille Université, CNRS Marseille 13288 France; ^3^ Laboratory of Molecular Neurobiology, Neurobiology Center Nencki Institute of Experimental Biology of the Polish Academy of Sciences Warsaw 02-093 Poland; ^4^ Molecular Biology and Nanotechnology (MolBNL@UniTS) Laboratory DEA University of Trieste Piazzale Europa 1 34127 Trieste Italy; ^5^ Department of Medicinal and Applied Chemistry Kaohsiung Medical University Kaohsiung 807 Taiwan; ^6^ Centre de Recherche en Cancérologie de Marseille (CRCM) INSERM U1068, CNRS UMR 7258 Aix-Marseille Université and Institut Paoli-Calmettes Marseille 13288 France; ^7^ Department of General Biophysics Faculty of Biology and Environmental Protection University of Lodz Łódź 90-236 Poland

**Keywords:** bola-amphiphiles, glycodendrimers, glucose transporters, mannose receptors, targeted delivery

## Abstract

Dendrimers are appealing scaffolds for creating carbohydrate mimics with unique multivalent cooperativity. We report here novel bola‐amphiphilic glycodendrimers bearing mannose and glucose terminals, and a hydrophobic thioacetal core responsive to reactive oxygen species. The peculiar bola‐amphiphilic feature enabled stronger binding to lectin compared to conventional amphiphiles. In addition, these dendrimers are able to target mannose receptors and glucose transporters expressed at the surface of cells, thus allowing effective and specific cellular uptake. This highlights their great promise for targeted delivery.

## Graphical abstract

**Smart dumbbell**: Innovative bola‐amphiphilic glycodendrimers with mannose and glucose terminals have been designed and synthesized. They demonstrated stronger binding to the carbohydrate‐binding protein ConA than conventional amphiphilic glycodendrimers thanks to their unique dumbbell‐like structure. In addition, these bola‐dendrimers targeted mannose receptors and glucose transporters for effective cellular uptake, and thus hold great promise in carbohydrate‐binding protein‐targeted drug delivery.

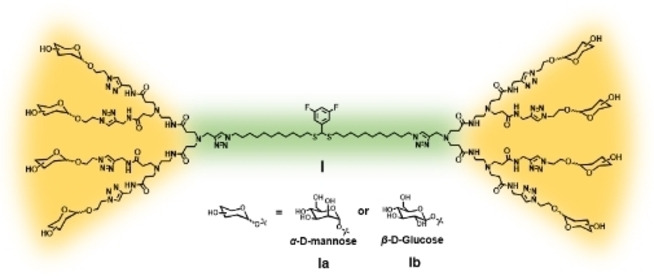

## Introduction

Carbohydrates are the most abundant biomacromolecules in living organisms and play important roles in energy production, as structural materials and as signaling moieties.[^1^, ^2^, ^3^, ^4^, ^5^] Carbohydrates mediate cell‐cell and cell‐matrix interactions through carbohydrate‐carbohydrate and carbohydrate‐protein binding.[^1^, ^2^, ^3^, ^4^, ^5^] Although single carbohydrate units often show low binding affinity, the glycoside cluster effect, which exploits the cooperative interactions issued from the branched and multivalent structure of carbohydrates, improves strength and specificity of binding.[^6^, ^7^, ^8^, ^9^] This particular effect is essential in cell‐cell interactions and in regulating various biological events, such as cell differentiation, proliferation and adhesion, inflammation, cancer metastasis and immune responses.[^1^, ^2^, ^3^, ^4^, ^5^]

Dendrimers, by virtue of their unique ramified structure and multivalent cooperativity, are particularly appealing scaffolds for constructing carbohydrate mimics.[^10^, ^11^, ^12^, ^13^, ^14^] Such dendrimers, also referred to as glycodendrimers, can structurally and functionally mimic natural polysaccharides, glycoproteins and mucins, and hence constitute a useful means for studying the glycoside cluster effect and for targeting specific carbohydrate‐binding proteins. Recently, Percec et al. developed Janus amphiphilic glycodendrimers that can self‐assemble into dendrimersomes, mimicking the recognition structures of glycans and glycoproteins on the cell.[^15^, ^16^, ^17^] In addition, amphiphilic dendrimers bearing carbohydrate modifications have been developed for drug delivery.[^18^, ^19^] For example, a photo‐responsive polypeptide‐glycosylated dendron amphiphile was documented to dynamically bind to concanavalin A (ConA) for ovalbumin (OVA) delivery.^[18]^ Also, a lactose‐bearing amphiphilic poly(amidoamine) (PAMAM) dendrimer was reported to effectively interact with RCA_120_ lectin and deliver the anticancer drug doxorubicin.^[19]^ Compared with many reported conventional glycodendrimer systems, amphiphilic glycodendrimers are able to self‐assemble and form dynamic systems, offering modular and adaptive interactions with carbohydrate‐binding proteins. Amphiphilic glycodendrimers therefore hold great potential in targeted drug delivery.

**Figure 1:**
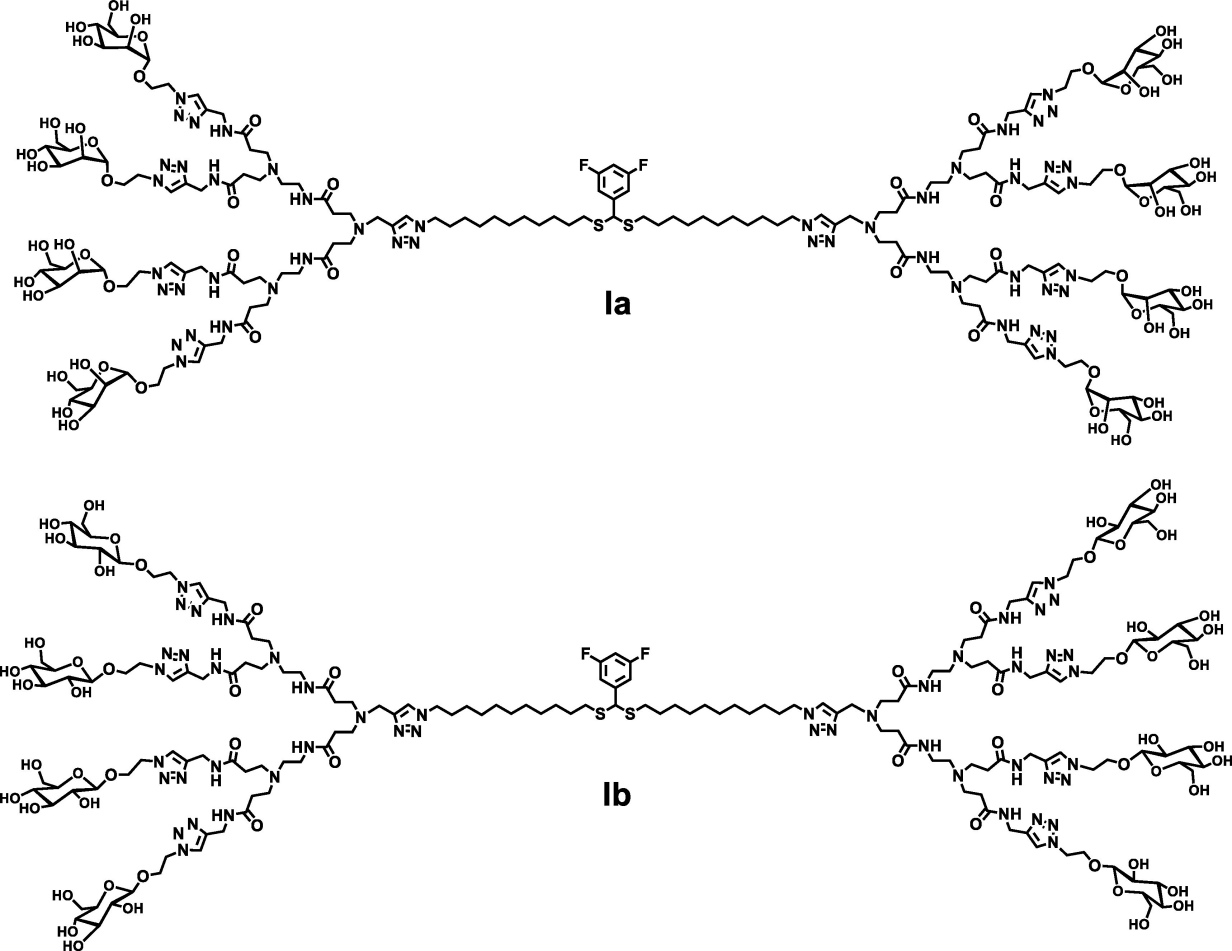
Bola‐amphiphilic glycodendrimers bearing mannose (**Ia**) and glucose (**Ib**) terminals.

In this study, we report the synthesis and the use of novel bola‐amphiphilic glycodendrimers (Figure 1) for binding and targeting specific carbohydrate‐binding proteins. Different from traditional amphiphilic molecules, the dumbbell‐like shape of bola‐amphiphiles offers a unique bipolar amphiphilic structure,[^20^, ^21^] which is expected to reinforce molecular recognition, binding and stability towards carbohydrate‐binding proteins. This approach should allow the exploitation of the glycoside cluster effect and the implementation of multivalent glycoconjugates for targeted delivery. In addition, the core of the bola‐amphiphilic glycodendrimers harbors a thioacetal functionality, which is responsive to reactive oxygen species (ROS), thus allowing redox‐responsive delivery.^[22]^

Specifically, in this work we sought to introduce mannose and glucose entities to the bola‐amphiphilic dendrimer terminals (Figure 1) and study their capacity to interact with carbohydrate‐binding proteins such as lectin ConA, the mannose receptor (MR) and the glucose transporter (GLUT). ConA is a tetramer lectin with a strong affinity for mannose residues, forming a crosslinked network or aggregations with mannosyl carbohydrates through multivalent and cooperative interactions.^[23]^ The MR can effectively bind mannose ligand and is mainly expressed on the surface of macrophages (including microglia‐brain‐resident myeloid cells), dendritic cells, endothelial cells and some cancer cells,[^24^, ^25^] while the GLUT is widely expressed in the blood‐brain barrier (BBB), astrocytes and cancer cells.[^26^, ^27^] The GLUT is mainly responsible for the “two‐way” transport of glucose that maintains a stable concentration of this carbohydrate in cells, and plays an important role in normal physiological balance.^[26]^ As such, the GLUT is often found overexpressed in cancer cells owing to their requirement of large quantities of glucose for rapid proliferation.^[27]^ Based on these considerations, we designed the bola‐amphiphilic glycodendrimers **Ia** and **Ib**, both carbohydrate mimics bearing respectively mannose and glucose terminals, studied their binding with ConA and their capacity to target specific cells overexpressing MRs and GLUTs.

## Results and Discussion

### Dendrimer synthesis

**Figure 2:**
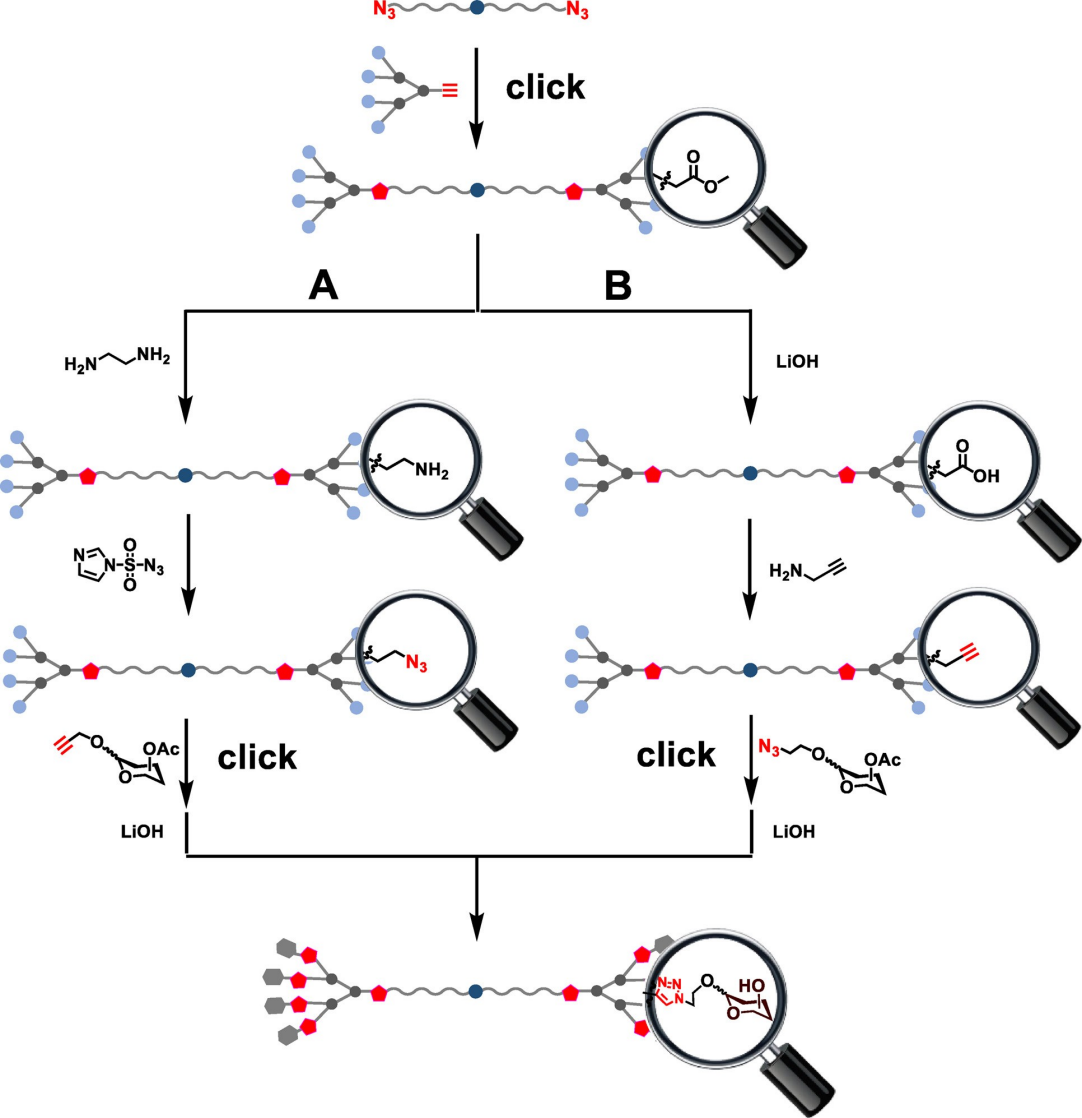
Strategies proposed for synthesizing the bola‐amphiphilic glycodendrimers.

For synthesizing the bola‐amphiphilic dendrimers **Ia** and **Ib** we envisaged two strategies (Figure 2), mainly exploiting “click” reactions to generate the bola‐amphiphilic dendrimer scaffold and to introduce the carbohydrate terminal units. Specifically, the ester‐bearing bola‐amphiphilic dendrimer would be first constructed in a click reaction by coupling the azido‐bearing hydrophobic chain with the alkyne‐containing hydrophilic PAMAM dendron; the obtained dendrimer would then be transformed to bear the azido or alkynyl terminals which would, through a second click reaction with the corresponding alkyne‐ and azide‐bearing carbohydrate precursors, deliver the bola‐amphiphilic glycodendrimers.

**Figure 3:**
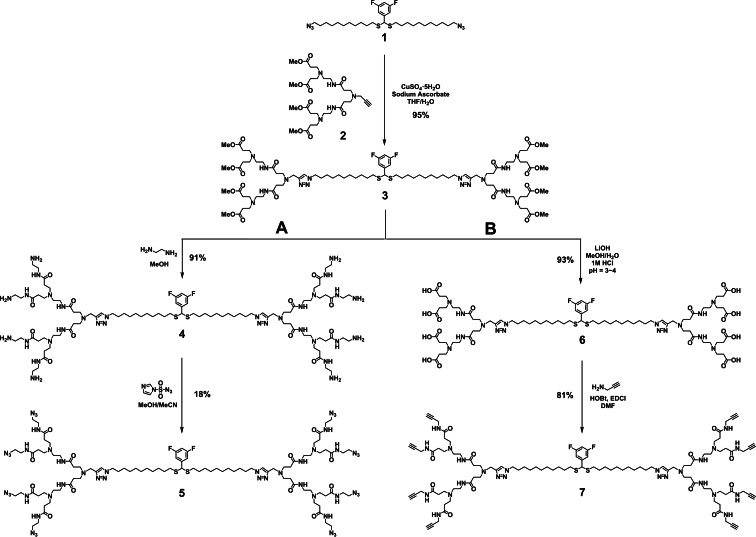
Synthesis of the intermediate dendrimers **5** and **7** required for the preparation of the glycodendrimers.

Accordingly, we first prepared the ester terminating dendrimer **3** using Cu(I)‐catalyzed 1,3‐dipolar cycloaddition to couple the hydrophobic chain **1** bearing two azido terminals with the alkyne‐containing PAMAM dendron **2** as reported (Figure 3).^[22]^ We then attempted strategy A, which started well with the successful conversion of **3** to the amine‐terminating dendrimer **4** upon treatment with ethylene diamine. However, the reaction to transform the terminal amino groups in **4** to azido functions was inefficient despite attempting various different conditions when using imidazole‐1‐sulfonyl azide as the amine‐azide transfer reagent.^[28]^ This reaction gave many side products, likely due to incomplete multi‐site reactions. In addition, purification of the azido‐terminating dendrimer **5** was very difficult.

We then turned to strategy B to synthesize alkynyl‐terminating dendrimer **7** (Figure 3). We first hydrolyzed the methyl ester terminated dendrimer **3** using LiOH to yield the carboxylic acid terminated dendrimer **6** within a short time (3 h). Then dendrimer **6** was conjugated with propargylamine using the coupling reagents EDCI and HOBt in DMF to generate the alkynyl‐terminating dendrimer **7**. Remarkably, this conjugation went very well, and thin layer chromatography (TLC) showed only one main product formed even though there were eight reaction sites in **6**. Dendrimer **7** was easily purified using column chromatography and was obtained at a high yield of 81 %.

**Figure 4:**
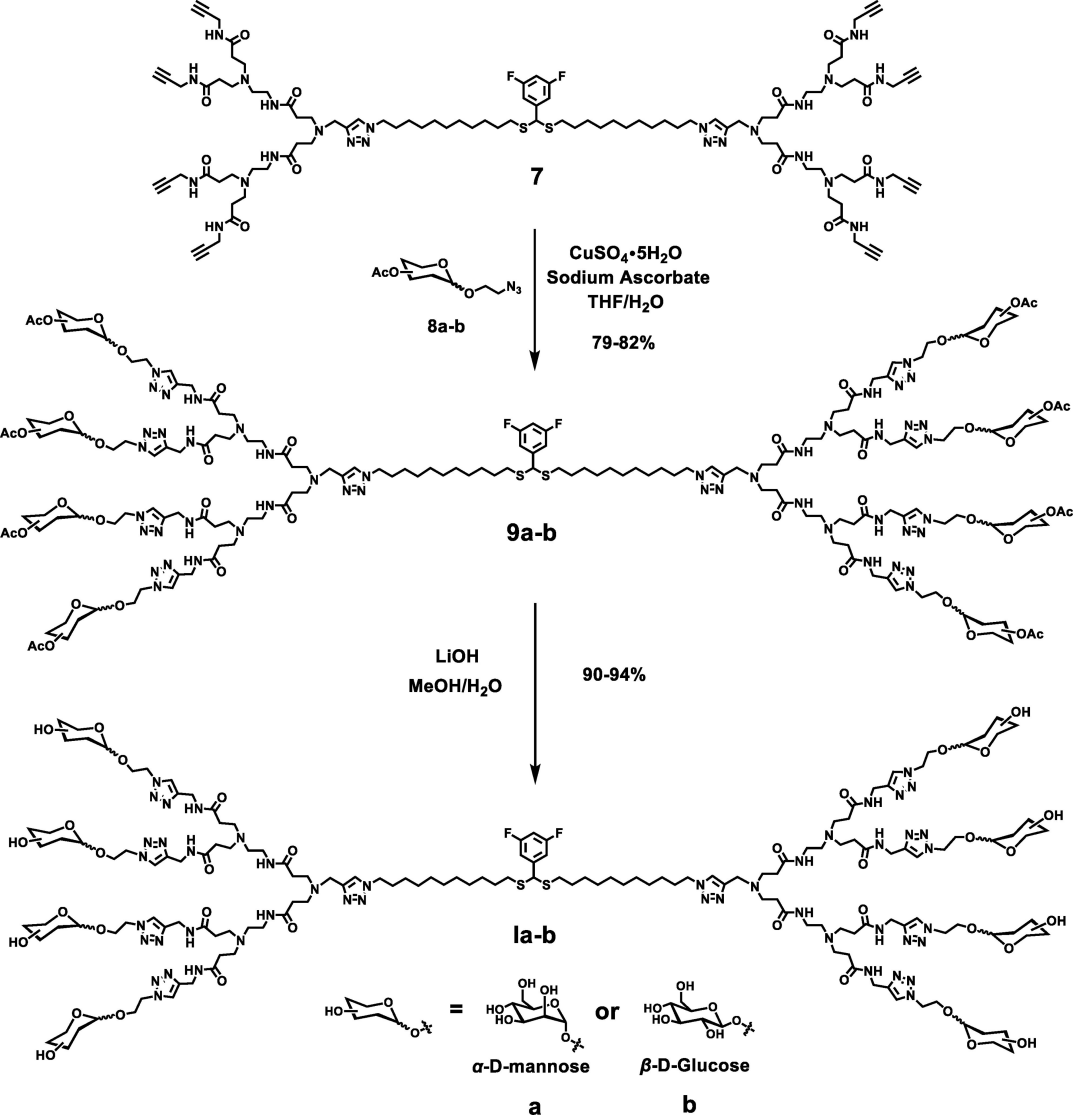
Synthesis of the bola‐amphiphilic glycodendrimers **Ia** and **Ib**.

Starting with **7**, the click reactions with the azido‐conjugated mannose and glucose precursors **8** **a**–**b**, respectively, were extremely successful (Figure 4) despite the existence of multi‐site reactions and steric hindrance among the carbohydrate terminals. The desired dendrimers **9** **a**–**b** were obtained with yields up to 80 % after column chromatography.

**Figure 5:**
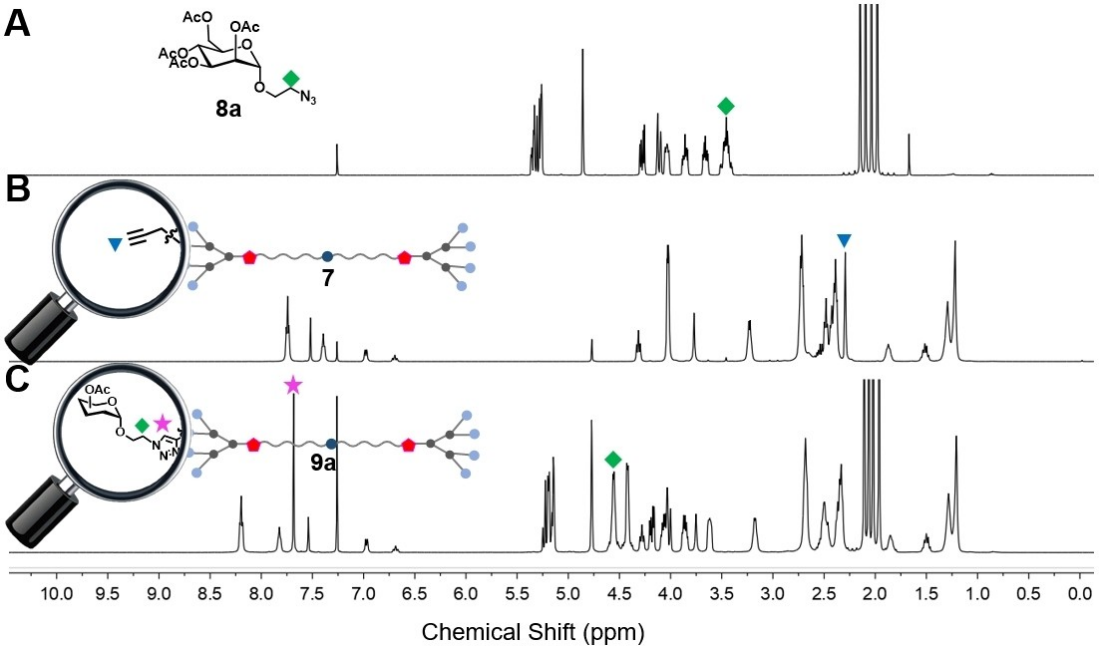
^1^H NMR spectral analysis of A) **8** **a**, B) **7** and C) **9** **a** in CDCl_3_ to confirm the successful synthesis of **9** **a** by click chemistry. The peaks marked with ⧫, ▾ and ★ belong to the characteristic NMR signals from protons of methylene linked to an azide group in **8** **a**, alkynyl terminals in **7** and triazole rings in the terminal part of **9** **a**, respectively.

NMR analysis of the transformation from dendrimer **7** to dendrimer **9** **a** is presented in Figure 5. The disappearance of the NMR peak at 2.30 ppm (labeled as blue triangle, corresponding to the alkynyl terminals in **7**) revealed that all the alkynyl terminals were fully consumed in the click reaction. Also, the newly generated triazole functions were indicated with the NMR peak at 7.68 ppm (labeled as pink star, corresponding to the protons of the eight triazole rings in the terminal part of **9** **a**). These NMR data confirmed the successful conjugation of the carbohydrate units at the dendrimer terminals (Figure 5B and C). It is noteworthy that the protons on the linker (labeled with green square) between monosaccharide and triazole moved to the low‐field owing to the deshielding effect of newly formed triazoles (Figure 5A and C).

Upon treatment with LiOH, the 32 acetyl groups in dendrimers **9** **a** and **9** **b** were successfully removed within 3 h, indicating that the large steric hindrance had little impact on the deprotection process. Both **Ia** and **Ib** were obtained at yields above 90 % after purification using dialysis. The structural integrity and purity of all the synthesized dendrimer final products and intermediates were confirmed using ^1^H, ^13^C, and ^19^F NMR spectral analysis as well as high‐resolution mass spectroscopy (HRMS; see the Supporting Information).

### Biocompatibility with no notable cytotoxicity

**Figure 6:**
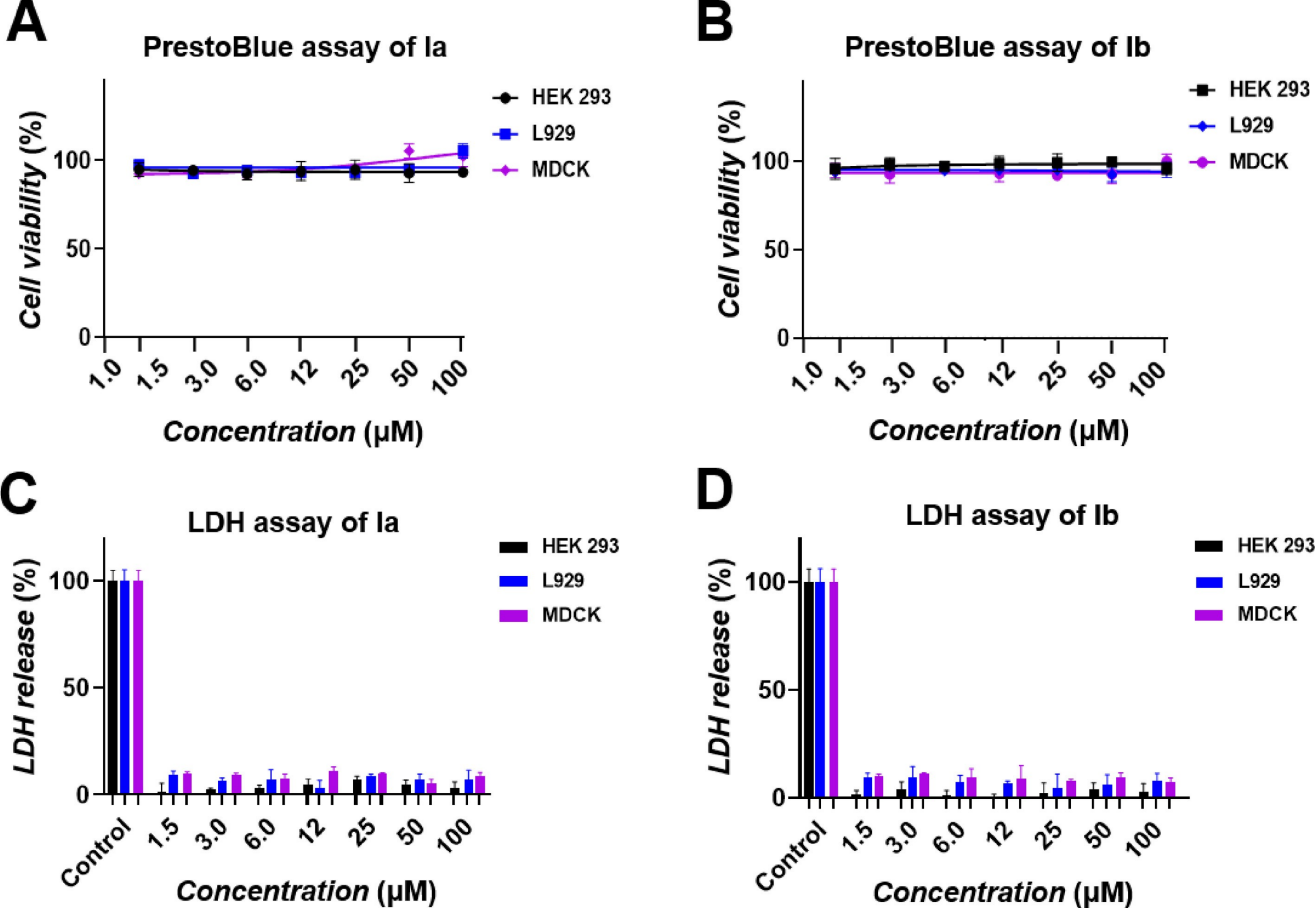
Cytotoxicity evaluation of A), C) **Ia** and B), D) **Ib** on HEK293, L929 and MDCK cells in a PrestoBlue assay and lactate dehydrogenase (LDH) test. Cells treated with lysis buffer served as control with 100 % LDH release; mean ± SD, *n*=3.

The bola‐amphiphilic dendrimers **Ia** and **Ib** are well solubilized in water at concentrations up to 30 mg/mL. In addition, both **Ia** and **Ib** showed good biocompatibility with no notable cytotoxicity, as revealed by the cytotoxicity data obtained with human embryonic kidney cells (HEK293), mouse fibroblast cells (L929), and Madin‐Darby canine kidney cells (MDCK) using PrestoBlue assay and lactate dehydrogenase (LDH) test (Figure 6). The PrestoBlue assay evaluates metabolic toxicity by measuring the cell viability, and the LDH test examines toxicity associated with cell membrane damage by determining the release of lactate dehydrogenase. Results from both the PrestoBlue and LDH assays (Figure 6) indicated that both **1** **a** and **1** **b** were devoid of any notable toxicity.

### Effective and strong binding to lectin

**Figure 7:**
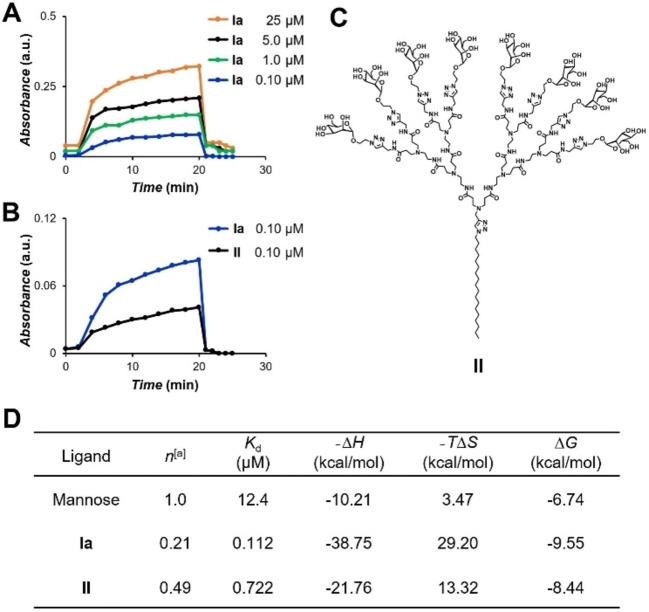
Binding of **Ia** to ConA shown in a turbidimetric assay and by isothermal titration calorimetry. Turbidity curves recorded at 450 nm for the precipitation of ConA with A) **Ia** at different concentrations, and B) **Ia** and **II** at 0.10 μM in HEPES buffer at room temperature. C) Chemical structure of the conventional amphiphilic dendrimer counterpart **II**. D) ITC thermodynamics parameters for ConA binding at 300 K. Errors are less than 8 % of the value. [a] Number of ligands/number of protein monomers.

As ConA has a strong affinity for α‐d‐mannosyl residues but not for β‐d‐glucosyl residues,[^23^, ^29^] we studied the binding of **Ia** with ConA using **Ib** as the negative control. Adding **Ia** to ConA immediately generated a white precipitate owing to the strong and multivalent interaction between **Ia** and ConA, whereas the ConA solution remained clear upon **Ib** addition, even at concentrations up to 500 μM. Using turbidimetric assay at 450 nm, we demonstrated that the binding between **Ia** and ConA was concentration dependent, and remained effective even at concentrations as low as 0.10 μM (Figure 7A). Moreover, **Ia** showed significantly stronger binding compared to the conventional amphiphilic dendrimer **II** bearing the same number of mannose terminals (Figure 7B, C and Figure S1 in the Supporting Information). These results highlight the important contribution of the unique bola‐amphiphilic structural feature of glycodendrimer **Ia** towards the observed stronger protein binding and higher glycoside cluster effect.

Further evaluation of the binding affinity and thermodynamics of the bola‐amphiphilic dendrimer **Ia** towards ConA was carried out using isothermal titration calorimetry (ITC), with the conventional amphiphilic dendrimer **II** serving as the control for comparison (Figure 7D). All the thermodynamics results are reported in (Figure 7D), while the raw thermograms and the integrated heats for both systems can be found in Figure S2. The thermodynamic signature of binding and the *K*_d_ values obtained for the single mannose molecule, used as control, agree with literature data.^[30]^ Furthermore, the number of detected binding sites (*n*) of 1.0, is consistent with the monovalent nature of the interaction of this carbohydrate with ConA. Regarding the newly synthesized glycodendrimers, the values of *n* <1 for both dendrimers corroborate the hypothesis of a multivalent nature in the binding between their terminal carbohydrates and lectin. Moreover, the high entropic penalty (−*T*Δ*S*) detected for both systems is in line with the possibility of a crosslinking mechanism between each dendrimer and the protein. According to the data reported in Figure 7D, **Ia** seems to exploit multivalency in a more effective way upon protein binding, as the corresponding observed value of *n* is closer to the ideal value of 0.125, whilst that obtained for the **II**/ConA complex is more than twofold larger. Finally, a further confirmation of the lectin binding assay results comes from the analysis of the thermodynamics patterns; indeed, **Ia** outperforms **II** with a more favorable Δ*G* value of −9.55 kcal/mol (vs. −8.44 kcal/mol, Figure 7D), corresponding to an affinity for ConA of 0.112 μM.

### Cell‐specific targeting

**Figure 8:**
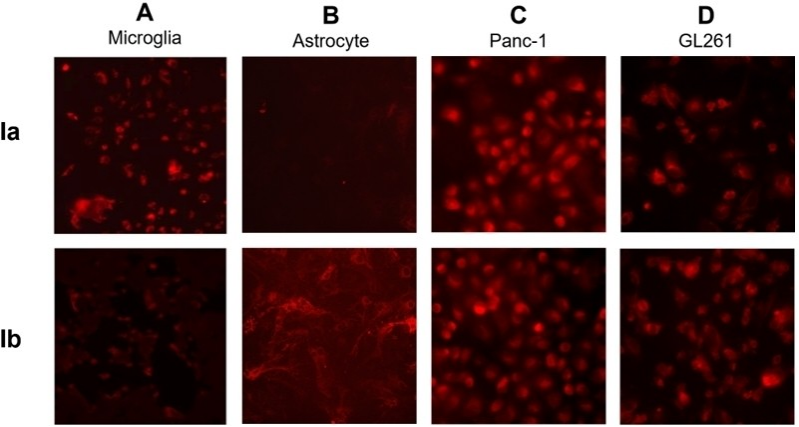
Fluorescent imaging of the cellular uptake of Cy5‐labeled glycodendrimers **Ia** and **Ib** in A) primary microglia cultures, B) primary astrocyte cultures, and of the Cy3‐labeled glycodendrimers **Ia** and **Ib** in C) human pancreatic cancer Panc‐1 cells and D) mouse glioblastoma GL261 cells.

We also assessed the targeting ability of **Ia** and **Ib** towards MRs and GLUTs by examining their cellular uptake in primary microglia and astrocyte cultures, respectively chosen for their overexpression of MRs and high levels of GLUTs. Notably, microglia internalized **Ia** more efficiently compared to **Ib** (Figure 8A), whereas astrocytes showed prominent cellular uptake of **Ib** but little of **Ia** (Figure 8B). These results demonstrate the effective and specific targeting shown by **Ia** to MRs on microglia and by **Ib** to GLUTs on astrocytes.

Considering the high cell surface expression of MRs and GLUTs shown by many cancers,^[27]^ we also evaluated the targeting ability of **Ia** and **Ib** towards human pancreatic cancer Panc‐1 cells and mouse glioma GL261 cells. Both **Ia** and **Ib** were effectively internalized by both cells (Figure 8C and D) in a concentration‐dependent manner (Figure S3). Collectively, these data highlight the cancer cell‐targeting ability of **Ia**‐**b** and their potential use for targeted delivery of anticancer drugs.

## Conclusion

In conclusion, we have established novel bola‐amphiphilic glycodendrimers with mannose and glucose terminals, and studied their properties with regards to binding and targeting specific carbohydrate‐binding proteins. These dendrimers were readily synthesized by using sequential click reactions and showed good water solubility and biocompatibility. The unique bola‐amphiphilic structure of **Ia** allowed its stronger binding to the lectin ConA compared to that of conventional amphiphilic glycodendrimer **II**. In addition, **Ia** specifically targeted the mannose receptor, whereas **Ib** specifically targeted the glucose transporter, and demonstrated effective cellular uptake in microglia and astrocytes, respectively, as well as cancer cells. Collectively, these bola‐amphiphilic glycodendrimers hold great promise for drug delivery targeted to cells expressing carbohydrate‐binding proteins; we are actively working in this direction.

## Experimental Section

**General information**: Compounds **1** and **2** were synthesized according to the protocol previously established.[^22^, ^31^] The carbohydrate derivatives **8** **a** and **8** **b** were synthesized using the reported methods.[^32^, ^33^] Chemicals were purchased from Sigma‐Aldrich or Alfa Aesar. Methyl acrylate, *N,N*‐dimethylethylenediamine, ethylenediamine, dichloromethane and methanol were dried using the described methods and distilled prior to use. The other chemicals were used without further purification. Concanavalin A (Con A) for carbohydrate binding studies was purchased from Sigma‐Aldrich. Dialysis tubing was purchased from Sigma‐Aldrich. Analytical thin layer chromatography (TLC) was performed using silica gel 60 F254 plates 0.2 mm thick with UV light (254 and 364 nm) as revelator. Chromatography was prepared on silica gel (Merck 200–300 mesh). ^1^H, ^13^C and ^19^F NMR spectra were recorded on a Bruker Avance III 400 at 400, 100 and 376 MHz, respectively. Chemical shifts (*δ*) are presented in parts per million (ppm) using the residual peak of CHCl_3_ at 7.26 ppm or the residual peak of CH_3_OH at 3.34 ppm as internal reference. The high‐resolution mass spectra (HRMS) were recorded on a SYNAPT G2 HDMS (Waters) mass spectrometer equipped with an electrospray ionization (ESI) source. The sample was ionized in positive electrospray mode under the following conditions: electrospray voltage: 2.8 kV; orifice voltage: 20 V; and nebulizer gas flow (nitrogen): 100 L/h.

**Synthesis of 6**: To a solution of compound **3** (180 mg, 0.10 mmol) in MeOH (10 mL) was added aqueous solution of LiOH (39 mg, 1.6 mmol) while stirring under the protection of argon. The reaction mixture was stirred at 25 °C for 3 h. The MeOH was removed by rotavapor and followed by the addition of 1 M HCl solution until the pH reached 4. The product was purified by dialysis using dialysis tube of MWCO 2000, followed by lyophilization. After repeating 4 times the operation of dialysis and lyophilization, the product was lyophilized to give compound **6** as a white solid (164 mg, 93 %).

**Synthesis of 7**: To the compound **6** (170 mg, 0.10 mmol) were added HOBT (250 mg, 1.6 mmol), EDCI (310 mg, 1.6 mmol) and propargylamine (88 mg, 1.6 mmol) in 15 mL anhydrous DMF. The vessel was sealed and purged with argon for 5 min. The reaction mixture was stirred at 25 °C for 72 h until the reaction was complete, as indicated by TLC analysis. The DMF was evaporated under reduced pressure and the resulting residue was suspended in NaHCO_3_ solution (30 mL). The water phase was extracted with CH_2_Cl_2_ (3 x 30 mL). The combined organic layers were dried over Na_2_SO_4_, filtered and concentrated. The resulting crude product was purified by column chromatography on silica gel to yield **7** as a slight yellow oil (190 mg, 81 %); *R*_f_ = 0.35 (CH_2_Cl_2_/MeOH 6 : 1).

**Synthesis of 9** **a**: To the compound **7** (100 mg, 0.050 mmol) were added CuSO_4_⋅5H_2_O (10 mg, 0.040 mmol), sodium ascorbate (16 mg. 0.080 mmol) and a solution of **8** **a** (250 mg, 0.60 mmol) in 12 mL THF. The vessel was sealed and purged with argon for 5 min and 3.0 mL H_2_O was then added into the mixture. The reaction mixture was stirred at 60 °C for 6 h until the reaction was complete, as indicated by TLC analysis. The THF was evaporated under reduced pressure and the resulting residue was suspended in EDTA solution (30 mL). The water phase was extracted with CH_2_Cl_2_ (3×30 mL). The combined organic layers were dried over Na_2_SO_4_, filtered and concentrated. The resulting crude product was purified by column chromatography on silica gel to yield **9** **a** as a slight yellow oil (210 mg, 79 %); *R*_f_ = 0.30 (CH_2_Cl_2_/MeOH 5 : 1).

**Synthesis of 9** **b**: To the compound **7** (100 mg, 0.050 mmol) were added CuSO_4_⋅5H_2_O (10 mg, 0.040 mmol), sodium ascorbate (16 mg. 0.080 mmol) and a solution of **8** **b** (250 mg, 0.60 mmol) in 12 mL THF. The vessel was sealed and purged with argon for 5 min and 3.0 mL H_2_O was then added into the mixture. The reaction mixture was stirred at 60 °C for 6 h until the reaction was complete, as indicated by TLC analysis. The THF was evaporated under reduced pressure and the resulting residue was suspended in EDTA solution (30 mL). The water phase was extracted with CH_2_Cl_2_ (3×30 mL). The combined organic layers were dried over Na_2_SO_4_, filtered and concentrated. The resulting crude product was purified by column chromatography on silica gel to yield **9** **b** as a slight yellow oil (220 mg, 82 %); *R*_f_ = 0.30 (CH_2_Cl_2_/MeOH 5 : 1).

**Synthesis of Ia**: To a solution of compound **9** **a** (110 mg, 0.020 mmol) in MeOH (12 mL) was added aqueous solution of LiOH (31 mg, 1.3 mmol) while stirring under argon. The reaction mixture was stirred at 25 °C for 3 h. The MeOH was removed by rotavapor and followed by the addition of 1 M HCl solution until the pH reached 7. The product was purified by dialysis using dialysis tube of MWCO 2000, followed by lyophilization. After repeating 4 times the operation of dialysis and lyophilization, the product was lyophilized to give compound **Ia** as a white solid (72 mg, 90 %).

**Synthesis of Ib**: To a solution of compound **9** **b** (110 mg, 0.020 mmol) in MeOH (12 mL) was added aqueous solution of LiOH (31 mg, 1.3 mmol) while stirring under the protection of argon. The reaction mixture was stirred at 25 °C for 3 h. The MeOH was removed by rotavapor and followed by the addition of 1 M HCl solution until the pH reached 7. The product was purified by dialysis using dialysis tube of MWCO 2000, followed by lyophilization. After repeating 4 times the operation of dialysis and lyophilization, the product was lyophilized to give compound **Ib** as a white solid (75 mg, 94 %).

**Turbidimetric assay**: Mannose dendrimers and Concanavalin A (Con A) binding was studied using turbidity assay by measuring the increasing absorbance at *λ*=450 nm after addition of mannose dendrimers to Con A solution. 1.0 mg/mL Con A solution in HEPES‐buffer (pH 7.0) was stirred for 2 h at room temperature and filtered through a 4 μm syringe filter. 1.0 mM mannose dendrimers stock solution was prepared in HEPES‐buffer. HEPES‐buffer solution was transferred into a quartz cuvette (1 cm cell length) and used as blank solution to set the absorbance as zero. Later Con A solution (1.9 mL) was transferred into a quartz cuvette and absorbance readings were collected every second at 450 nm. After 2 min a specific volume from the respective mannose dendrimer stock solution added in Con A solution mixed vigorously for 2 s using a micropipette to get desired dendrimer final concentration. The absorbance was recorded at 450 nm for 18 min and then the inhibition assay was performed by adding α‐methyl mannose solution (20 μL at 10 mg/mL). Absorbance data were recorded at 450 nm for 5 min.

**Isothermal titration calorimetry measurements**: ITC experiments were conducted using a MicroCal PEAQ‐ITC calorimeter (Malvern Ltd.) at 300 K. The cell volume was 208 μL. All experiments were conducted by step‐by‐step injections of a constant volume of ligand (Mannose, **Ia** or **II**) solution into the calorimetric cell containing a solution of ConA in 0.10 M HEPES buffer containing NaCl (0.90 %), CaCl_2_ (0.10 mM) and MnCl_2_ (0.10 mM) at pH 7.2. Specifically, after a pre‐rinse injection of 0.40 L, the ligand solution was injected in 18 portions of 2.0 μL at 150 s intervals. All solutions were degassed for 30 min at room temperature under stirring at 750 rpm prior to each experiment. After washing, the cell was prerinsed with a portion of the simple buffer solution and upon filling the cell and syringe, stirring was turned on and each system was allowed to thermally equilibrate for 30 min. The heat signals resulting from mixing, dilution effects and liquid friction were further confirmed by control experiments (data not shown); accordingly, they were subtracted from the relevant datasets to yield the corrected integrated data. All experiments were run in triplicate.

**Cells**: Primary microglia cultures were prepared from the cerebral cortices of P0‐P2 C57BL6 J mouse pups as previously described.^[34]^ Microglial cells were isolated from glial culture monolayers by gentle shaking, then collected by centrifugation, checked for viability and seeded at required density in high‐glucose culture medium DMEM supplemented with Glutamax, 10 % fetal bovine serum (ThermoScientific; CA, USA) and antibiotics. The remaining glial cell culture monolayer was left with strong shaking at 180 rpm overnight to remove sparse oligodendrocytes and receive >97 % pure astrocyte culture.

Human pancreatic cancer cells Panc‐1, human embryonic kidney 293 cells (HEK 293 cells), mouse fibroblast cells (L929 cells), Madin‐Darby canine kidney cells (MDCK cells) and Murine glioma GL261 cells were cultured in Dulbecco's modified Eagle's medium (DMEM) (Gibco, Invitrogen) containing 10 % fetal bovine serum (FBS; Biosera). Cells were maintained at 37 °C with 5 % CO_2_ in a humidified chamber.

**PrestoBlue assay**: HEK 293, L929/MDCK and Panc‐1/GL261 cells were seeded at 1.0×10^4^ cells/well, 4.0×10^3^ cells/well and 5.0×10^3^ cells/well in 96‐well plates, and all the cells were allowed to grow overnight. Cells were then treated with various concentration (1.0–100 μM) of the bola‐amphiphilic dendrimer for 72 h. Then 10 μL PrestoBlue reagent was added to each well containing 100 μL of blank, control, or treated cells in culture and incubated for another 1–3 h at 37 °C. The changes in cell viability were detected using fluorescence spectroscopy (*λ*_ex_=570 nm; *λ*_em_=610 nm). The cell viability was expressed as a percentage relative to the cells untreated with dendrimers. All samples were run in triplicate.

**Lactate dehydrogenase (LDH) assay**: The different cell lines were seeded in 96‐well plates and cultured overnight. The density of HEK293 cells was 1.0×10^4^ cells per well. The density of MDCK cells and L929 cells was 4.0×10^3^ cells per well. Then, HEK293, MDCK and L929 cells were respectively treated with various concentrations (1.0–100 μM) of dendrimer for 24 h. Afterwards, cell membrane damage toxicity was determined using CytoTox‐ONE Homogeneous Membrane Integrity Assay (Promega). The LDH reaction mixture was freshly prepared according to the manufacturer's protocol, 100 μL added to each well of a 96‐well plate containing 100 μL of blank, control or treated cells. The cells were incubated for 30 min at 25 °C followed by adding 50 μL of stop solution. The optical density (OD) of these solutions were measured at 490 nm by fluorescence spectroscopy. Control was performed with lysis buffer and medium, and set as 100 % and 0 % LDH release, respectively. Each assay was performed in triplicate.

LDH%=[(the absorbance of sample−the absorbance of negative control)/(the absorbance of positive control−the absorbance of negative control)]×100 %.

**Cellular uptake**: Visualization of cells for analyzing cellular uptake was performed using fluorescence microscopes.

Microglia were seeded at a density of 2.0–3.0×10^5^ cells/cm^2^ onto glass culture slides and astrocytes were plated on poly‐l‐lysine‐coated glass cover slips in 24‐well culture plates at a density of 1.0–1.5×10^5^ cells/cm^2^ two days before transfection. The cells were incubated with Cy5‐labeled bola‐amphiphilic glycodendrimers (4.0 μM) for 3 h at 37 °C. Next the medium was removed, the cells were washed twice with PBS and the microphotographs were taken using Olympus IX70 microscope.

Panc‐1 and GL261 cells were respectively seeded in 12‐well plate (1.0×10^5^ cells/well) one day before transfection. The cells were incubated with Cy3‐labeled bola‐amphiphilic glycodendrimers (2.0 and 4.0 μM) for 3 h at 37 °C. The cells were washed with cold PBS buffer, and then Hoechst33342 (1.0 μg/mL) was added, and stained for 30 min at 37 °C. A Zeiss Apotome.2 microscope (Carl Zeiss Microscopy GmbH, Goettingen, Germany) was used for visualization. Images were acquired using ZEN 3.1 pro software (Carl Zeiss GmbH).

## Conflict of interest

The authors declare no conflict of interests.

1

## Supporting information

As a service to our authors and readers, this journal provides supporting information supplied by the authors. Such materials are peer reviewed and may be re‐organized for online delivery, but are not copy‐edited or typeset. Technical support issues arising from supporting information (other than missing files) should be addressed to the authors.

Supporting Information

## Data Availability

The data that support the findings of this study are available in the supplementary material of this article.
