# Supplementary material for: Bola‐Amphiphilic Glycodendrimers: New Carbohydrate‐Mimicking Scaffolds to Target Carbohydrate‐Binding Proteins
Source: Chemistry. 2022 Aug 26;28(58):e202201400. doi: 10.1002/chem.202201400 (PMC9804701; doi:10.1002/chem.202201400)
Supplement: Supplementary file 1 — Supporting Information [file CHEM-28-0-s001.pdf]

Chemistry–A European Journal

Supporting Information

Bola-Amphiphilic Glycodendrimers: New Carbohydrate-Mimicking Scaffolds to Target Carbohydrate-Binding Proteins

Wenzheng Zhang, Dinesh Dhumal, Xiaolei Zhu, Brigino Ralahy, Aleksandra Ellert-Miklaszewska, Jing Wu, Erik Laurini, Yi-Wen Yao, Chai-Lin Kao, Juan L. Iovanna, Sabrina Pricl, Bozena Kaminska, Yi Xia,* and Ling Peng*

Table of Contents

Figure S1. .... S2

Figure S2. .... S3

Figure S3. .... S4

Experimental section S5

NMR spectra of compounds.. S19

Reference.. S32

Figure S1. Synthesis of dendrimer **II**.

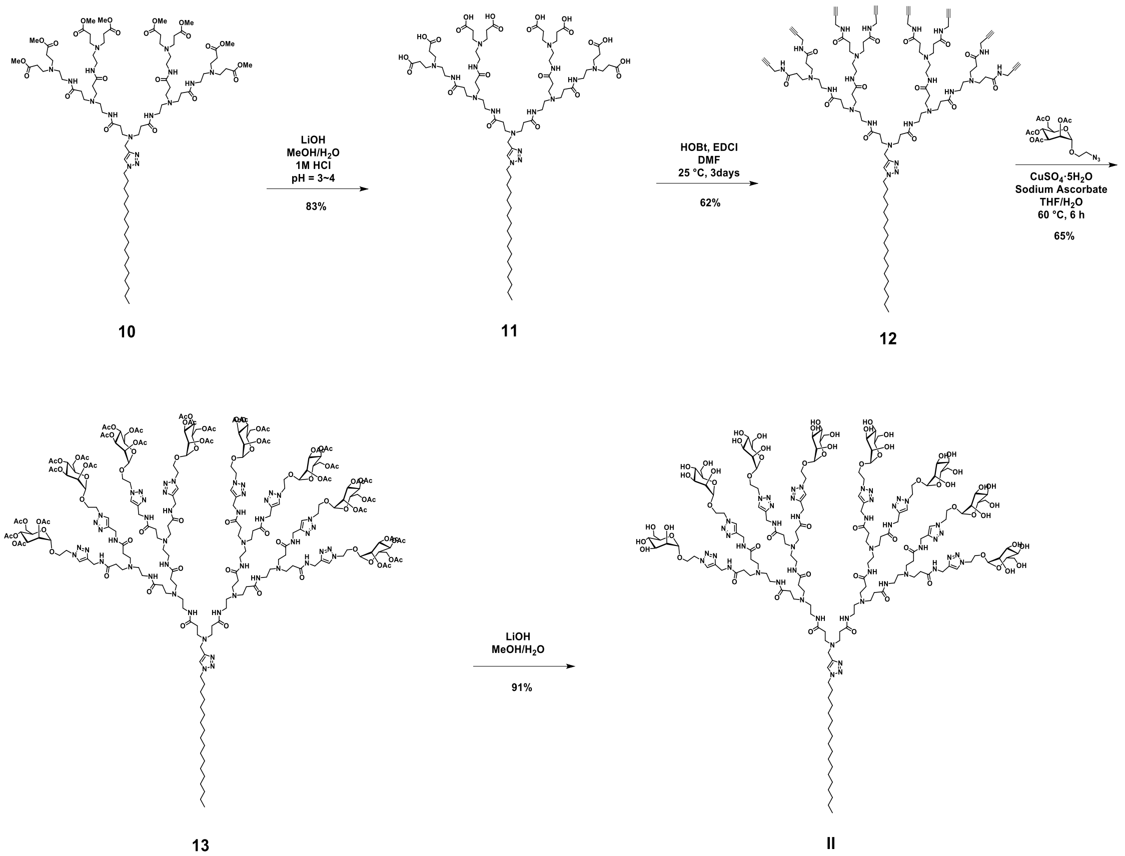

Figure S2. Isothermal titration calorimetry raw integrated data for titration of ConA with **Ia** (top panel) and **II** (bottom panel) in buffer (T = 300 K). The insets are the corresponding raw thermograms. The solid line represents data fitting with a sigmoidal function.

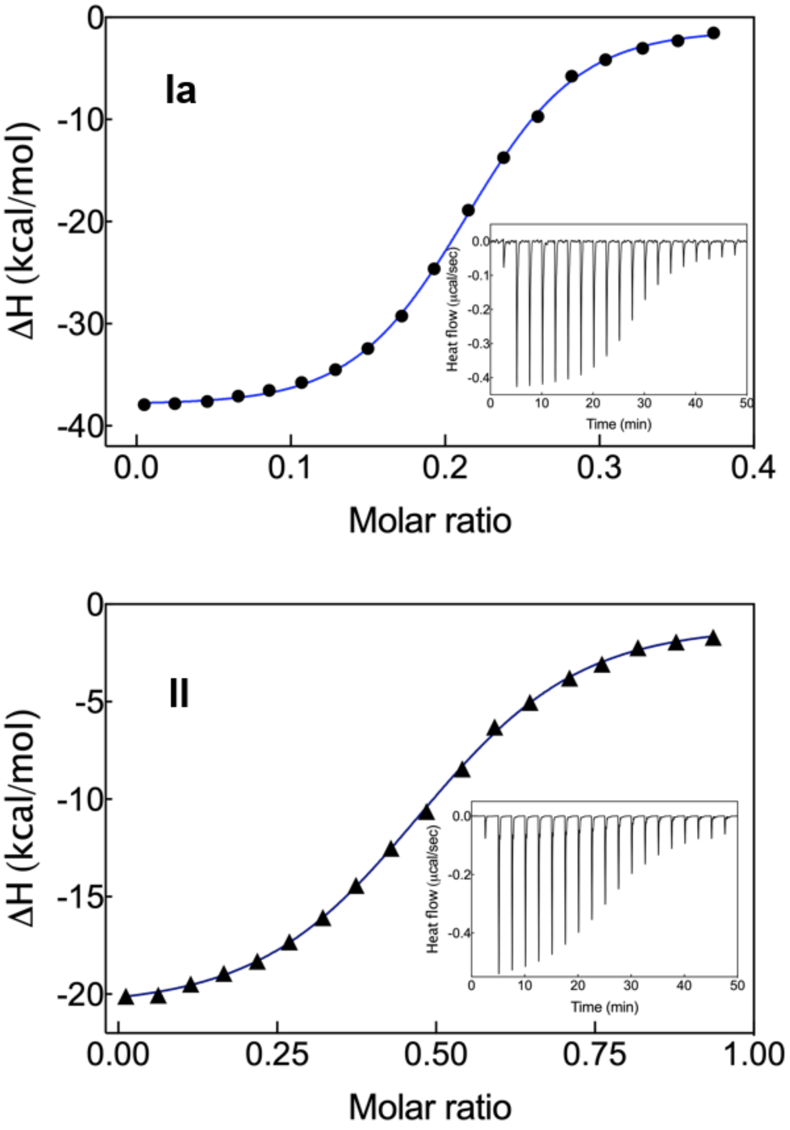

Figure S3. Cell uptake of **Ia** and **Ib** in (A) human pancreatic cancer Panc-1 cells and (B) mouse glioma GL261 cells assessed using fluorescent microscopy with Cy3-labelled dendrimers.

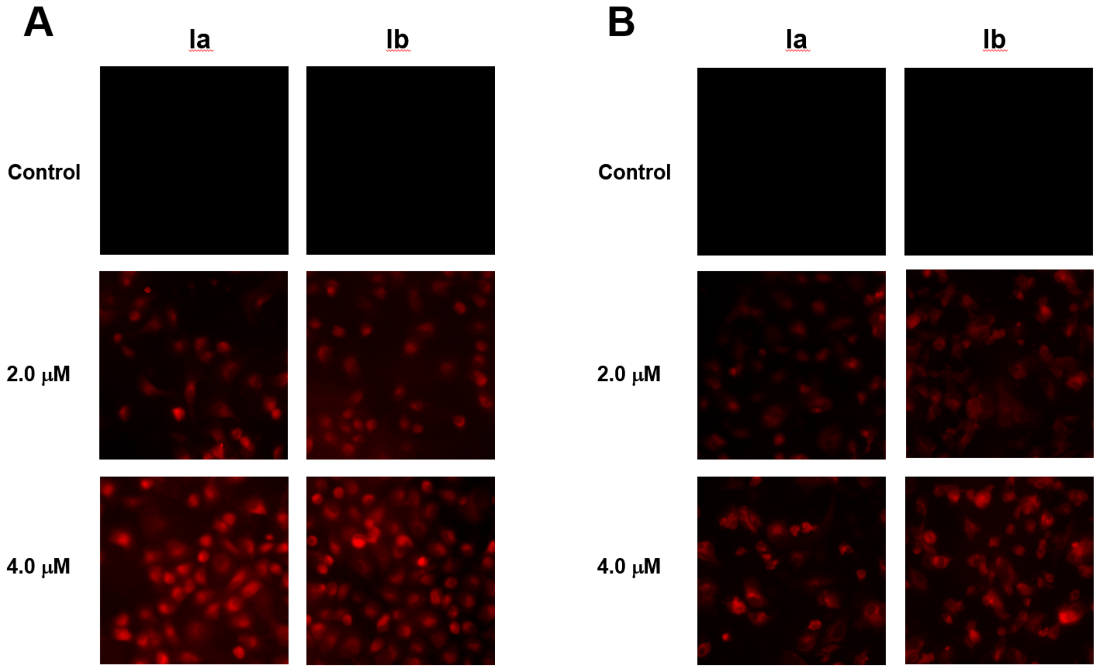

Experimental section

Synthesis and characterization of compound 3

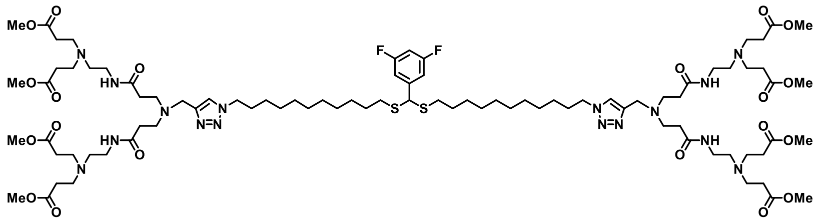

To the azide precursor **1** (290 mg, 0.50 mmol) were added $CuSO_4·5H_2O$ (25 mg, 0.10 mmol), sodium ascorbate (40 mg, 0.20 mmol) and a solution of **2** (780 mg, 1.3 mmol) in 12 mL THF. The vessel was sealed and purged with argon for 5 min and 3.0 mL H_2O was then added into the mixture. The reaction mixture was stirred at 60 °C for 90 min until the reaction was complete, as indicated by TLC analysis. The THF was evaporated under reduced pressure and the resulting residue was suspended in EDTA solution (30 mL). The water phase was extracted with CH_2Cl_2 (10 mL×3). The combined organic layers were dried over Na_2SO_4, filtered and concentrated. The resulting crude product was purified by column chromatography on silica gel to yield **3** as a colorless oil (870 mg, 95%); R_f= 0.25 (EtOAc/MeOH 2:1).

^{1}H-NMR (400 MHz, $CDCl_3$) δ 7.50 (s, 2H, 2×CH), 7.11 (t, 4H, J = 5.1 Hz, 4×NH), 7.00-6.95 (m, 2H, ArH), 6.71-6.65 (m, 1H, ArH), 4.76 (s, 1H, CH), 4.29 (t, 4H, J = 7.36 Hz, 2×CH_2), 3.81 (s, 4H, 2×CH_2), 3.64 (s, 24H, 8×CH_3), 3.26 (q, 8H, J =5.64 Hz, 4×CH_2), 2.77 (t, 8H, J = 6.6 Hz, 4×CH_2), 2.73 (t, 16H, J = 6.7 Hz, 8×CH_2), 2.58-2.46 (m, 12H, 6×CH_2), 2.43-2.39 (m, 24H, 12×CH_2), 1.89-1.82 (m, 4H, 2×CH_2), 1.54-1.47 (m, 4H, 2×CH_2), 1.32-1.21 (m, 28H, 14×CH_2). ^{13}C-NMR (100 MHz, $CDCl_3$) δ 173.14, 172.18, 164.22, 164.10, 161.75, 161.62, 145.05, 144.96, 144.88, 143.90, 122.57, 110.93, 110.86, 110.74, 110.67, 103.57, 103.32, 103.07, 53.03, 52.54, 51.74, 50.34, 49.34, 47.76, 37.17, 33.74, 32.78, 32.41, 30.42, 29.52, 29.48, 29.45, 29.21, 29.09, 28.89, 26.06. ^{19}F-NMR (376 MHz, $CDCl_3$) δ -109.17. HRMS: calcd. for $C_{87}H_{146}F_2N_{16}O_{20}S_2$ $[M+4H]^{4+}$ 460.5157, found 460.5148.

Synthesis of characterization of compound 4

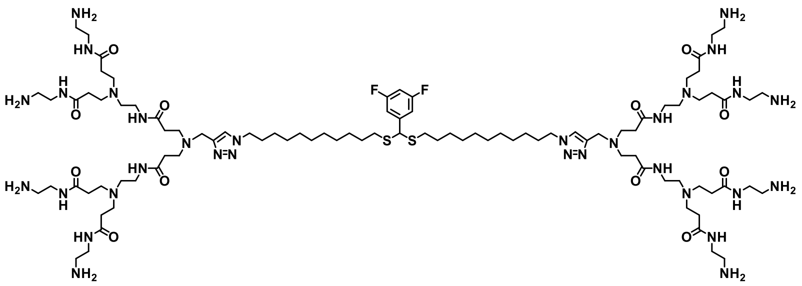

To a solution of **3** (96 mg, 0.054 mmol) in MeOH (8.0 mL) was added ethylenediamine (560 μL, 8.1 mmol). The reaction mixture was stirred under argon for 72 h at 30 °C. The reaction solution was evaporated, and the obtained residue was purified by dialysis with a dialysis tube (MWCO=1000). Subsequent lyophilisation yielded compound **4** as white powder (96 mg, 91%).

^{1}H-NMR (400 MHz, CD_3OD) δ 7.94 (s, 2H, 2×CH), 7.10 (d, 2H, J = 7.6 Hz, ArH), 6.88 (t, 1H, J = 8.9 Hz, ArH), 5.02 (s, 1H, CH), 4.42 (t, 4H, J = 6.9 Hz, 2×CH_2), 3.84 (s, 4H, 2×CH_2), 3.28 (t, 24H, J = 6.2 Hz, 12×CH_2), 2.83-2.73 (m, 40H, 20×CH_2), 2.66-2.52 (m, 12H, 6×CH_2), 2.45 (t, 8H, J = 6.4 Hz, 4×CH_2), 2.39 (t, 16H, J = 6.5 Hz, 8×CH_2), 1.96-1.89 (m, 4H, 2×CH_2), 1.61-1.53 (m, 4H, 2×CH_2), 1.38-1.29 (m, 28H, 14×CH_2); ^{13}C-NMR (100 MHz, CD_3OD) δ 175.20, 174.67, 165.57, 165.45, 163.11, 162.98, 147.53, 147.45, 147.36, 144.81, 125.15, 111.99, 111.92, 111.80, 111.73, 104.11, 103.85, 103.59, 53.43, 53.22, 51.34, 51.16, 50.39, 48.44, 42.95, 42.02, 38.57, 34.81, 34.64, 33.33, 31.33, 30.53, 30.18, 30.09, 29.76, 27.52; ^{19}F-NMR (376 MHz, CD_3OD) δ -111.26; HRMS: calcd. for $C_{95}H_{178}F_2N_{32}O_{12}S_2$ $[M+4H]^{4+}$ 516.6007, found 516.6006.

Synthesis of characterization of compound 5

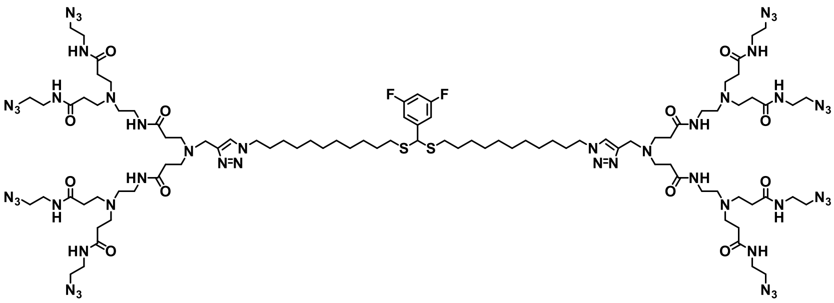

To a solution of azidosulfonyl imidazole (140 mg, 0.80 mmol) in CH_3CN (12 mL) and CH_3OH (3.0 mL) were added **4** (100 mg, 0.050 mmol), $CuSO_4·5H_2O$ (10 mg, 0.040 mmol) and K_2CO_3 (110 mg, 0.80 mmol). The resulting mixture was stirred at room temperature for 24 h. The solvent was evaporated under vacuum and followed by the addition of H_2O (30 mL). The resulting mixture was extracted with ethyl acetate (50 mL×3). Then the organic phase was combined, dried with $MgSO_4$, filtered and concentrated to get a crude product. The resulting crude product was purified by column chromatography on silica gel to yield **5** as a slight yellow oil (33 mg, 18%); R_f = 0.40 (CH_2Cl_2/MeOH 6:1).

^{1}H-NMR (400 MHz, $CDCl_3$) δ 7.59 (m, 14H, 12×NH, 2×CH), 6.99 (m, 2H, ArH), 6.71 (m, 1H, ArH), 4.78 (s, 1H, CH), 4.32 (t, 4H, J = 7.5 Hz, 2×CH_2), 3.78 (s, 4H, 2×CH_2), 3.42 (m, 32H, 16×CH_2), 3.26 (q, 8H, J = 5.9 Hz, 4×CH_2), 2.76-2.73 (m, 24H, 12×CH_2), 2.58-2.48 (m, 12H, 6×CH_2), 2.43-2.38 (m, 24H, 12×CH_2), 1.90-1.86 (m, 4H, 2×CH_2), 1.56-1.49 (m, 4H, 2×CH_2), 1.34-1.23 (m, 28H, 14×CH_2);^{13}C-NMR (100 MHz, $CD_3OD/CDCl_3$) δ 174.51, 173.92, 164.91, 164.78, 162.44, 162.31, 146.00, 143.91, 124.34, 111.45, 111.19, 103.91, 103.65, 103.40, 52.89, 51.05, 50.66, 47.86, 42.51, 41.54, 38.04, 34.35, 34.01, 32.93, 30.88, 30.04, 30.01, 29.69, 29.62, 29.33, 27.09; ^{19}F-NMR (376 MHz, $CD_3OD/CDCl_3$) δ -110.04.

Synthesis and characterization of compound 6

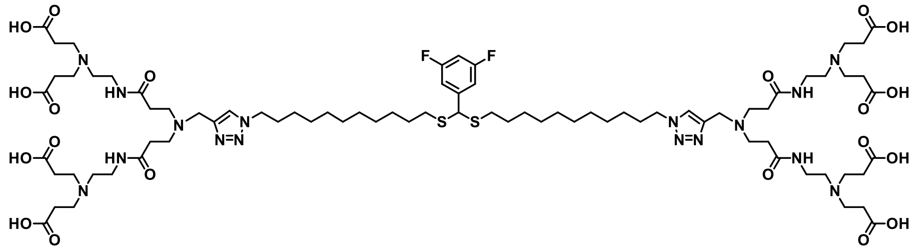

To a solution of compound **3** (180 mg, 0.10 mmol) in MeOH (10 mL) was added aqueous solution of LiOH (39 mg, 1.6 mmol) while stirring under the protection of argon. The reaction mixture was stirred at 25 °C for 3 h. The MeOH was removed by rotavapor and followed by the addition of 1 M HCl solution until the pH = 4. The product was purified by dialysis using dialysis tube of MWCO 2000, followed by lyophilization. After repeating 4 times the operation of dialysis and lyophilization, the

product was lyophilized to give compound **6** as a white solid (164 mg, 93%).

^{1}H-NMR (400 MHz, CD_3OD) δ 8.21 (s, 2H, 2×CH), 6.98 (d, 2H, J = 7.6 Hz, ArH), 6.77 (t, 1H, J = 8.84 Hz, ArH), 4.90 (s, 1H, CH), 4.40 (s, 4H, 2×CH_2), 4.35 (t, 4H, J = 6.96 Hz, 2×CH_2), 3.56 (t, 8H, J = 5.16 Hz, 4×CH_2), 3.30-3.25 (m, 32H, 16×CH_2), 2.77 (t, 8H, J = 6.72 Hz, 4×CH_2), 2.59 (t, 16H, J = 6.24 Hz, 8×CH_2), 2.53-2.40 (m, 4H, 2×CH_2), 1.86-1.79 (m, 4H, 2×CH_2), 1.50-1.42 (m, 4H, 2×CH_2), 1.28-1.18 (m, 28H, 14×CH_2). ^{13}C-NMR (100 MHz, CD_3OD) δ 176.66, 173.39, 165.57, 165.44, 163.10, 162.97, 147.38, 138.07, 128.12, 111.99, 111.74, 104.15, 103.89, 103.63, 53.19, 53.07, 51.50, 50.00, 35.52, 33.31, 31.66, 31.28, 30.79, 30.59, 30.55, 30.18, 30.14, 29.76, 27.52. ^{19}F-NMR (376 MHz, CD_3OD) δ -111.17. HRMS: calcd. for $C_{79}H_{130}F_2N_{16}O_{20}S_2$ $[M+2H]^{2+}$ 863.4601, found 863.4600.

Synthesis and characterization of compound 7

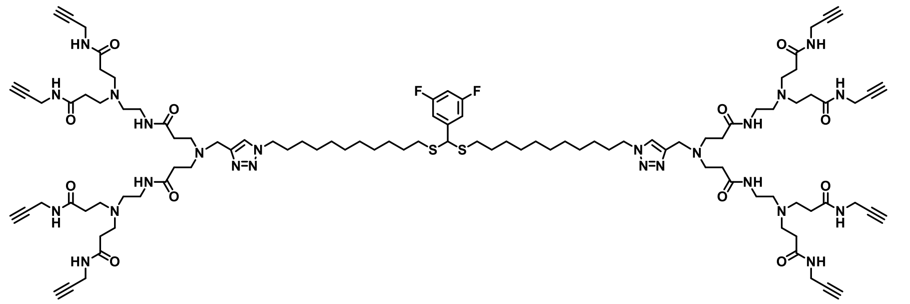

To the compound **6** (170 mg, 0.10 mmol) were added HOBT (250 mg, 1.6 mmol), EDCI (310 mg, 1.6 mmol) and propargylamine (88 mg, 1.6 mmol) in 15 mL anhydrous DMF. The vessel was sealed and purged with argon for 5 min. The reaction mixture was stirred at 25°C for 72 h until the reaction was complete, as indicated by TLC analysis. The DMF was evaporated under reduced pressure and the resulting residue was suspended in $NaHCO_3$ solution (30 mL). The water phase was extracted with CH_2Cl_2 (30 mL×3). The combined organic layers were dried over Na_2SO_4, filtered and concentrated. The resulting crude product was purified by column chromatography on silica gel to yield **7** as a slight yellow oil (190 mg, 81%); R_f = 0.35 (CH_2Cl_2/MeOH 6:1).

^{1}H-NMR (400 MHz, $CDCl_3$) δ 7.66 (t, 8H, J = 5.32 Hz, 8×NH), 7.53 (s, 2H, 2×CH), 7.33 (t, 4H, J = 5.4 Hz, 4×NH), 7.02-6.96 (m, 2H, ArH), 6.73-6.68 (m, 1H, ArH), 4.78

(s, 1H, CH), 4.32 (t, 4H, $J = 7.32$ Hz, 2×CH_2), 4.04 (dd, 16H, $J_a = 5.44$ Hz, $J_b = 2.56$ Hz, 8×CH_2), 3.79 (s, 4H, 2×CH_2), 3.24 (q, 8H, $J = 5.92$ Hz, 4×CH_2), 2.77-2.72 (m, 24H, 12×CH_2), 2.58-2.47 (m, 12H, 6×CH_2), 2.44 (t, 8H, $J = 6.16$ Hz, 4×CH_2), 2.40 (t, 16H, $J = 5.8$ Hz, 8×CH_2), 2.30 (t, 8H, $J = 2.48$ Hz, 8×CH), 1.92-1.85 (m, 4H, 2×CH_2), 1.57-1.49 (m, 4H, 2×CH_2), 1.34-1.23 (m, 28H, 14×CH_2). ^{13}C-NMR (100 MHz, $CDCl_3$) δ 172.86, 172.50, 164.30, 164.17, 161.82, 161.69, 145.11, 145.00, 144.92, 143.48, 143.45, 123.07, 111.00, 110.93, 110.81, 110.74, 103.65, 103.40, 103.15, 80.36, 71.49, 53.09, 52.61, 50.75, 50.56, 49.29, 47.67, 38.07, 34.33, 33.72, 32.46, 30.46, 29.56, 29.51, 29.24, 29.13, 29.07, 28.92, 26.66. ^{19}F-NMR (376 MHz, $CDCl_3$) δ -109.12. HRMS: calcd. for $C_{103}H_{154}N_{24}O_{12}F_2S_2$ $[M+4H]^{4+}$ 506.5477, found 506.5475.

Synthesis and characterization of compound 9a

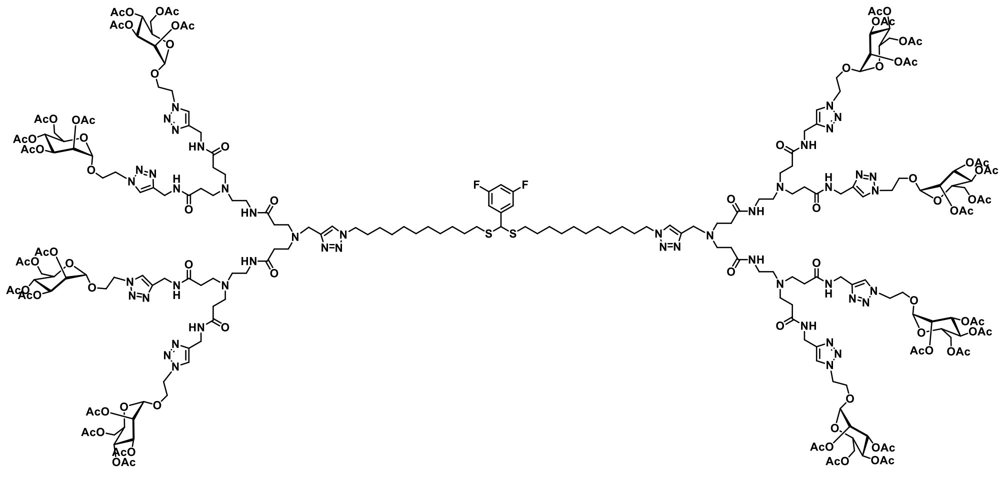

To the compound **7** (100 mg, 0.050 mmol) were added $CuSO_4{\cdot}5H_2O$ (10 mg, 0.040 mmol), sodium ascorbate (16 mg. 0.080 mmol) and a solution of **8a** (250 mg, 0.60 mmol) in 12 mL THF. The vessel was sealed and purged with argon for 5 min and 3.0 mL H_2O was then added into the mixture. The reaction mixture was stirred at 60 °C for 6 h until the reaction was complete, as indicated by TLC analysis. The THF was evaporated under reduced pressure and the resulting residue was suspended in EDTA solution (30 mL). The water phase was extracted with CH_2Cl_2 (30 mL×3). The combined organic layers were dried over Na_2SO_4, filtered and concentrated. The resulting crude product was purified by column chromatography on silica gel to yield

9a as a slight yellow oil (210 mg, 79%); R_f= 0.30 (CH_2Cl_2/MeOH 5:1).

^{1}H-NMR (400 MHz, $CDCl_3$) δ 8.20 (t, 8H, J = 5.28 Hz, 8×NH), 7.82 (t, 4H, J = 5.56 Hz, 4×NH), 7.68 (s, 8H, 8×CH), 7.54 (s, 2H, 2×CH), 6.97 (d, 2H, J = 6.8 Hz, ArH), 6.69 (t, 1H, J = 8.88 Hz, ArH), 5.25-5.13 (m, 24H, 24×CH), 4.77 (s, 9H, 9×CH), 4.61-4.54 (m, 16H, 8×CH_2), 4.51-4.37 (m, 16H, 8×CH_2), 4.28 (t, 4H, J = 7.44 Hz, 2×CH_2), 4.20-4.16 (m, 8H, 8×CH), 4.09-4.00 (m, 16H, 8×CH_2), 3.88-3.83 (m, 8H, 8×CH_2), 3.75 (s, 4H, 2×CH_2), 3.64-3.60 (m, 8H, 8×CH_2), 3.17 (q, 8H, J = 6.16 Hz, 4×CH_2), 2.68 (t, 24H, J = 6.00 Hz, 12×CH_2), 2.55-2.44 (m, 12H, 6×CH_2), 2.37 (t, 8H, J = 6.36 Hz, 4×CH_2), 2.33 (t, 16H, J = 6.28 Hz, 8×CH_2), 2.11 (s, 24H, 8×CH_3), 2.06 (s, 24H, 8×CH_3), 2.02 (s, 24H, 8×CH_3), 1.96 (s, 24H, 8×CH_3), 1.90-1.80 (m, 4H, 2×CH_2), 1.54-1.46 (m, 4H, 2×CH_2), 1.28-1.21 (m, 28H, 14×CH_2). ^{13}C-NMR (100 MHz, $CDCl_3$) δ 172.66, 172.49, 170.53, 169.92, 169.61, 164.07, 163.95, 161.60, 161.47, 145.27, 143.54, 123.34, 122.80, 110.80, 110.73, 110.61, 110.54, 103.43, 103.18, 102.93, 97.40, 69.07, 68.82, 66.15, 65.61, 62.11, 52.85, 52.39, 50.54, 50.22, 49.56, 47.33, 37.57, 34.56, 33.97, 33.27, 32.28, 30.30, 29.59, 29.41, 29.36, 29.08, 28.96, 28.76, 26.50, 20.79, 20.69, 20.6. ^{19}F-NMR (376 MHz, $CDCl_3$) δ -109.16. HRMS: calcd. for $C_{231}H_{338}F_2N_{48}O_{92}S_2$ $[M+5H]^{5+}$ 1073.2620, found 1073.2620.

Synthesis and characterization of compound 9b

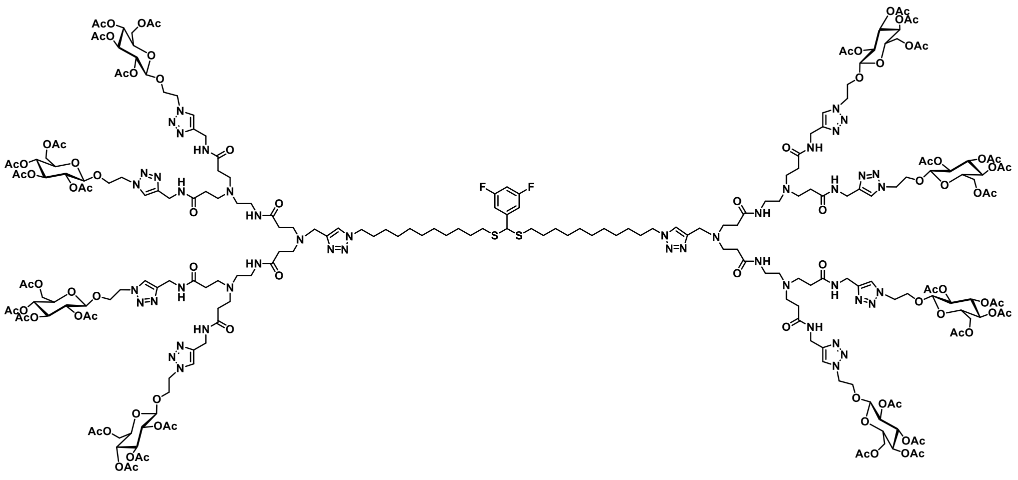

To the compound **7** (100 mg, 0.050 mmol) were added $CuSO_4 \cdot 5H_2O$ (10 mg, 0.040 mmol), sodium ascorbate (16 mg. 0.080 mmol) and a solution of **8b** (250 mg, 0.60

mmol) in 12 mL THF. The vessel was sealed and purged with argon for 5 min and 3.0 mL H_2O was then added into the mixture. The reaction mixture was stirred at 60 °C for 6 h until the reaction was complete, as indicated by TLC analysis. The THF was evaporated under reduced pressure and the resulting residue was suspended in EDTA solution (30 mL). The water phase was extracted with CH_2Cl_2 (30 mL×3). The combined organic layers were dried over Na_2SO_4, filtered and concentrated. The resulting crude product was purified by column chromatography on silica gel to yield **9b** as a slight yellow oil (220 mg, 82%); R_f = 0.30 (CH_2Cl_2/MeOH 5:1).

^{1}H-NMR (400 MHz, $CDCl_3$) δ 8.26 (t, 8H, *J* = 5.72 Hz, 8×NH), 7.84 (t, 4H, *J* = 5.2 Hz, 4×NH), 7.55 (s, 8H, 8×CH), 7.50 (s, 2H, 2×CH), 6.93 (d, 2H, *J* = 6.2 Hz, ArH), 6.65 (t, 1H, *J* = 8.84 Hz, ArH), 5.11 (t, 8H, *J* = 9.44 Hz, 8×CH), 5.00 (t, 8H, *J* = 9.76 Hz, 8×CH), 4.90 (t, 8H, *J* = 8.72 Hz, 8×CH), 4.72 (s, 1H, CH), 4.54-4.39 (m, 24H, 8×CH_2, 8×CH), 4.34 (d, 16H, *J* = 5.56 Hz, 8×CH_2), 4.26-4.11 (m, 20H, 10×CH_2), 4.06 (d, 8H, *J* = 12.04 Hz, 8×CH), 3.88-3.83 (m, 8H, 8×CH_2), 3.71 (s, 4H, 2×CH_2), 3.68-3.63 (m, 8H, 8×CH_2), 3.13 (q, 8H, *J* = 5.32 Hz, 4×CH_2), 2.66-2.61 (m, 24H, 12×CH_2), 2.53-2.40 (m, 12H, 6×CH_2), 2.34 (t, 8H, *J* = 6.52 Hz, 4×CH_2), 2.29 (t, 16H, *J* = 6.12 Hz, 8×CH_2), 2.02 (s, 24H, 8×CH_3), 1.95 (s, 24H, 8×CH_3), 1.93 (s, 24H, 8×CH_3), 1.90 (s, 24H, 8×CH_3), 1.85-1.76 (m, 4H, 2×CH_2), 1.50-1.42 (m, 4H, 2×CH_2), 1.25-1.17 (m, 28H, 14×CH_2). ^{13}C-NMR (100 MHz, $CDCl_3$) δ 173.55, 165.95, 165.74, 161.70, 161.68, 144.66, 143.37, 124.16, 112.06, 112.03, 111.98, 103.24, 76.75, 73.64, 70.17, 67.80, 61.35, 52.14, 51.80, 50.31, 49.80, 49.03, 37.21, 34.35, 33.22, 32.29, 31.97, 30.01, 29.24, 29.21, 28.84, 28.78, 28.42, 26.41, 26.20. ^{19}F-NMR (376 MHz, $CDCl_3$) δ -109.14. HRMS: calcd. for $C_{231}H_{338}F_2N_{48}O_{92}S_2$ $[M+5H]^{5+}$ 1073.2620, found 1073.2620.

Synthesis and characterization of compound Ia

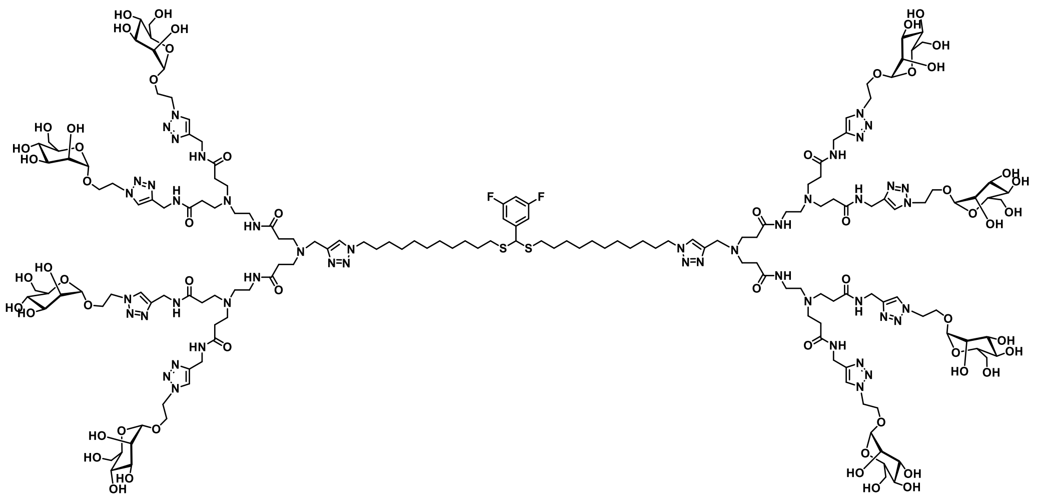

To a solution of compound **9a** (110 mg, 0.020 mmol) in MeOH (12 mL) was added aqueous solution of LiOH (31 mg, 1.3 mmol) while stirring under the protection of argon. The reaction mixture was stirred at 25 °C for 3 h. The MeOH was removed by rotavapor and followed by the addition of 1 M HCl solution until the pH = 7. The product was purified by dialysis using dialysis tube of MWCO 2000, followed by lyophilization. After repeating 4 times the operation of dialysis and lyophilization, the product was lyophilized to give compound **Ia** as a white solid (72 mg, 90%).

^{1}H-NMR (400 MHz, CD_3OD) δ 7.93 (s, 2H, 2×CH), 7.91 (s, 8H, 8×CH), 7.07 (d, 2H, *J* = 7.84 Hz, ArH), 6.85 (t, 1H, *J* = 9.6 Hz, ArH), 4.99 (s, 1H, CH), 4.71 (s, 8H, 8×CH), 4.60 (t, 16H, *J* = 5.64 Hz, 8×CH_2), 4.42 (s, 16H, 8×CH_2), 4.38 (t, 4H, *J* = 7.24 Hz, 2×CH_2), 4.10-4.04 (m, 8H, 8×CH), 3.85-3.81 (m, 12H, 8×CH, 2×CH_2), 3.73 (d, 16H, *J* = 10.88 Hz, 8×CH_2), 3.65-3.55 (m, 24H, 24×CH), 3.23 (t, 8H, *J* = 6.76 Hz, 4×CH_2), 3.06-2.99 (m, 8H, 8×CH), 2.80 (t, 16H, *J* = 6.84 Hz, 8×CH_2), 2.74 (t, 8H, *J* = 7.16 Hz, 4×CH_2), 2.63-2.49 (m, 12H, 6×CH_2), 2.43 (t, 8H, *J* = 6.76 Hz, 4×CH_2), 2.40 (t, 16H, *J* = Hz, 7.04, 8×CH_2), 1.92-1.85 (m, 4H, 2×CH_2), 1.57-1.50 (m, 4H, 2×CH_2), 1.31-1.26 (m, 28H, 14×CH_2). ^{13}C-NMR (100 MHz, CD_3OD) δ 173.63, 173.53, 167.38, 167.04, 163.32, 163.02, 146.48, 144.86, 124.85, 124.01, 112.76, 112.72, 103.91, 103.70, 103.47, 100.11, 73.53, 71.14, 70.59, 67.04, 65.25, 61.43, 49.94, 49.74, 49.03, 37.18, 34.36, 33.15, 31.99, 30.01, 29.20, 28.84, 28.41, 26.21. ^{19}F-NMR (376 MHz, CD_3OD) δ -111.28. HRMS: calcd. for $C_{167}H_{274}F_2N_{48}O_{60}S_2$ $[M+5H]^{5+}$ 804.1938, found 804.1932.

Synthesis and characterization of compound Ib

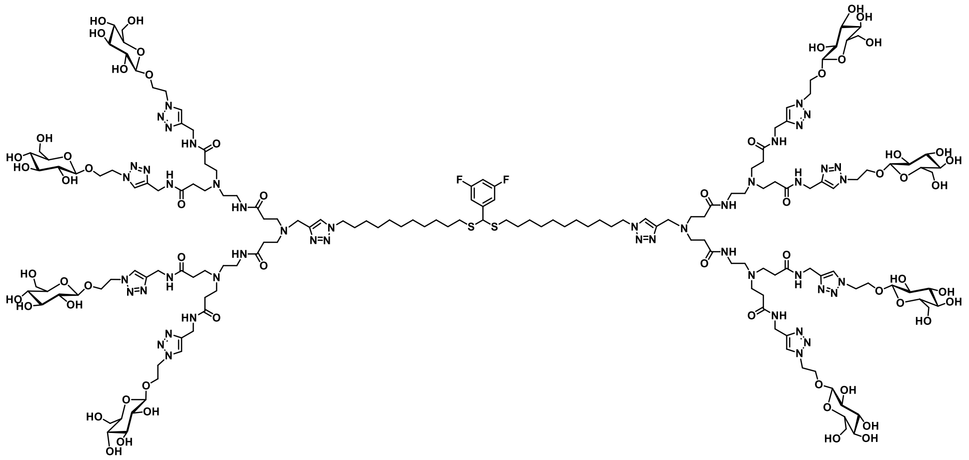

To a solution of compound **9b** (110 mg, 0.020 mmol) in MeOH (12 mL) was added aqueous solution of LiOH (31 mg, 1.3 mmol) while stirring under the protection of argon. The reaction mixture was stirred at 25 °C for 3 h. The MeOH was removed by rotavapor and followed by the addition of 1 M HCl solution until the pH = 7. The product was purified by dialysis using dialysis tube of MWCO 2000, followed by lyophilization. After repeating 4 times the operation of dialysis and lyophilization, the product was lyophilized to give compound **Ib** as a white solid (75 mg, 94%).

^{1}H-NMR (400 MHz, CD_3OD) δ 8.00 (s, 8H, 8×CH), 7.92 (s, 2H, 2×CH), 7.07 (d, 2H, J = 7.56 Hz, ArH), 6.85 (t, 1H, J = 8.56 Hz, ArH), 4.99 (s, 1H, CH), 4.60 (t, 16H, J = 5.36 Hz, 8×CH_2), 4.41-4.35 (m, 20H, 10×CH_2), 4.30 (d, 8H, J = 7.68 Hz, 8×CH), 4.23-4.17 (m, 8H, 8×CH_2), 4.00-3.94 (m, 8H, 8×CH_2), 3.86 (d, 8H, J = 11.92 Hz, 8×CH_2), 3.80 (s, 4H, 2×CH_2), 3.65 (d, 8H, J = 10.68 Hz, 8×CH_2), 3.35-3.33 (m, 8H, 8×CH), 3.30-3.27 (m, 8H, 8×CH), 3.23-3.15 (m, 16H, 16×CH), 2.80-2.71 (m, 24H, 12×CH_2), 2.61-2.51 (m, 12H, 6×CH_2), 2.42 (t, 8H, J = 6.56 Hz, 4×CH_2), 2.37 (t, 16H, J = 6.76 Hz, 8×CH_2), 1.92-1.86 (m, 4H, 2×CH_2), 1.57-1.50 (m, 4H, 2×CH_2), 1.40-1.26 (m, 28H, 14×CH_2). ^{13}C-NMR (100 MHz, CD_3OD) δ 173.55, 144.66, 124.16, 103.24, 76.75, 73.64, 70.17, 67.80, 61.35, 52.14, 51.80, 50.31, 49.80, 49.03, 37.21, 34.35, 33.22, 32.29, 31.97, 30.01, 29.24, 29.21, 28.84, 28.78, 28.42, 26.41, 26.20. ^{19}F-NMR (376 MHz, CD_3OD) δ -111.28. HRMS: calcd. for $C_{167}H_{274}F_2N_{48}O_{60}S_2$ $[M+4H]^{4+}$ 1004.9904, found 1004.9911.

Synthesis and characterization of compound 11

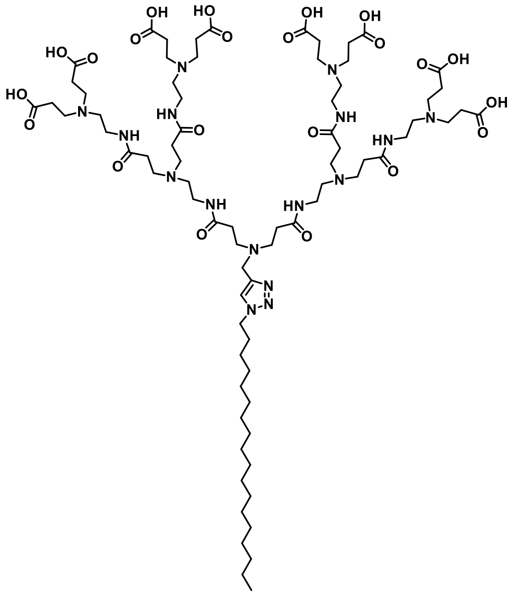

Compound 10 was synthesized according to the protocol previously established[1]. To a solution of **10** (300 mg, 0.17 mmol) in MeOH (5.0 mL) in ice bath, was slowly added LiOH·H_2O (120 mg, 2.8 mmol) in H_2O (5.0 mL). Then the solution was stirred at 25 °C for 3 h. When the reaction was completed indicated by NMR monitoring, MeOH was evaporated and the pH of the aqueous phase was adjusted to 3-4 using 1.0 M HCl solution, then purified by dialysis (dialysis tubing, MWCO 1000, changing dialysis water every hour for 6 times) and lyophilization. Repeating the operation cycles of dialysis and lyophilization 3 times to give **11** as a white solid (230 mg, 83%).

^{1}H-NMR (400 MHz, CD_3OD) δ 8.01 (s, 1H), 4.32 (t, 2H, J = 7.2 Hz), 4.11 (s, 2H), 3.57-3.53 (m, 12H), 3.35 (t, 8H, J = 6.8 Hz), 3.29-3.23 (m, 24H), 3.19 (t, 4H, J = 6.6 Hz), 2.97 (t, 4H, J = 6.9 Hz), 2.70 (t, 8H, J = 6.7 Hz), 2.60-2.54 (m, 20H), 1.85-1.78 (m, 2H), 1.24-1.18 (m, 30H), 0.80 (t, 3H, J = 6.3 Hz); ^{13}C-NMR (100 MHz, CD_3OD) δ 177.21, 173.87, 173.31, 140.88, 126.76, 53.79, 52.95, 51.77, 51.44, 50.62, 50.12, 47.62, 35.57, 33.07, 32.45, 31.47, 31.34, 31.06, 30.79, 30.71, 30.62, 30.47, 30.16, 27.56, 23.74, 14.47.; HRMS: calcd. for $C_{75}H_{134}N_{16}O_{22}$ $[M+3H]^{3+}$ 538.0026, found 538.0031.

Synthesis and characterization of compound 12

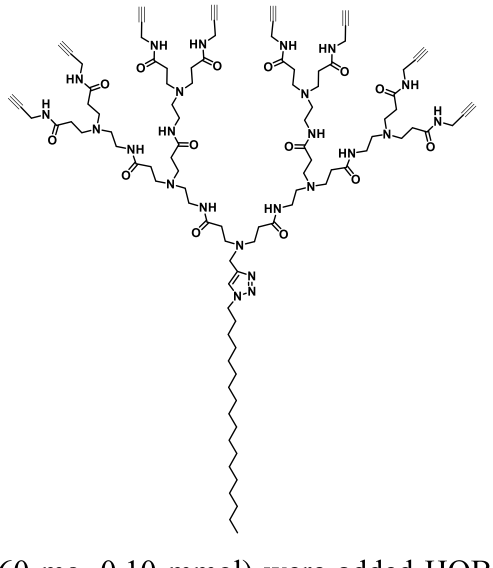

To the compound **11** (160 mg, 0.10 mmol) were added HOBT (250 mg, 1.6 mmol), EDCI (310 mg, 1.6 mmol) and propargylamine (88 mg, 1.6 mmol) in 15 mL anhydrous DMF. The vessel was sealed and purged with argon for 5 min. The reaction mixture was stirred at 25°C for 72 h until the reaction was complete, as indicated by TLC analysis. The DMF was evaporated under reduced pressure and the resulting residue was suspended in $NaHCO_3$ solution (30 mL). The water phase was extracted with CH_2Cl_2 (30 mL×3). The combined organic layers were dried over Na_2SO_4, filtered and concentrated. The resulting crude product was purified by column chromatography on silica gel to yield **12** as a slight yellow oil (120 mg, 62%); R_f = 0.30 (CH_2Cl_2/MeOH 6:1).

^{1}H-NMR (400 MHz, $CDCl_3$) δ 8.06 (t, 2H, J = 5.3 Hz, 2×NH), 7.81 (t, 8H, J = 5.4 Hz, 8×NH), 7.58 (s, 1H, CH), 7.34 (t, 4H, J = 5.7 Hz, 4×NH), 4.32 (t, 2H, J = 7.4 Hz, CH_2), 4.02 (m, 16H, 8×CH_2), 3.83 (s, 2H, CH_2), 3.25 (q, 12H, J = 6.2 Hz, 4×CH_2), 2.78-2.70 (m, 28H, 14×CH_2), 2.60-2.49 (m, 12H, 6×CH_2), 2.45 (t, 4H, J = 6.3 Hz, 2×CH_2), 2.41-2.38 (m, 24H, 12×CH_2), 2.32 (br.s, 8H, 8×CH), 1.91-1.84 (m, 2H, CH_2), 1.31-1.24 (m, 30H, 15×CH_2), 0.86 (t, 3H, J = 5.9 Hz, CH_3); ^{13}C-NMR (100 MHz, $CDCl_3$) δ 172.89, 172.81, 143.06, 123.19, 80.36, 71.55, 52.88, 52.54, 50.57, 50.45, 49.30, 47.10, 47.05, 37.80, 34.14, 34.06, 33.58, 32.03, 30.48, 29.81, 29.76, 29.70, 29.58, 29.47, 29.17, 29.06, 26.69, 22.80, 14.25; HRMS: calcd. for $C_{99}H_{158}N_{24}O_{14}$ $[M+4H]^{4+}$ 478.0677, found 478.0673.

Synthesis and characterization of compound 13

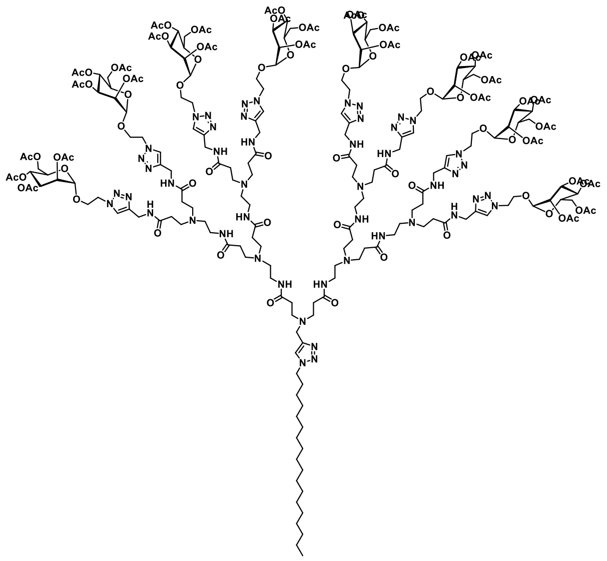

To the compound **12** (95 mg, 0.050 mmol) were added $CuSO_4·5H_2O$ (10 mg, 0.040 mmol), sodium ascorbate (16 mg. 0.080 mmol) and a solution of **8a** (250 mg, 0.60 mmol) in 12 mL THF. The vessel was sealed and purged with argon for 5 min and 3.0 mL H_2O was then added into the mixture. The reaction mixture was stirred at 60 °C for 6 h until the reaction was complete, as indicated by TLC analysis. The THF was evaporated under reduced pressure and the resulting residue was suspended in EDTA solution (30 mL). The water phase was extracted with CH_2Cl_2 (30 mL×3). The combined organic layers were dried over Na_2SO_4, filtered and concentrated. The resulting crude product was purified by column chromatography on silica gel to yield **13** as a slight yellow oil (170 mg, 65%); R_f= 0.30 (CH_2Cl_2/MeOH 5:1).

^{1}H-NMR (400 MHz, $CDCl_3$) δ 8.18 (t, 8H, J = 5.7 Hz, 8×NH), 7.93 (br.s, 2H, 2×NH), 7.80 (t, 4H, J = 6.2 Hz, 4×NH), 7.69 (s, 8H, 8×CH), 7.62 (s, 1H, CH), 5.24-5.13 (m, 24H, 24×CH), 4.77 (d, 8H, J = 1.72 Hz, 8×CH), 4.62-4.50 (m, 16H, 8×CH_2), 4.45-4.36 (m, 16H, 8×CH_2), 4.28 (t, 2H, J = 7.2 Hz, CH_2), 4.18 (dd, 8H, J_1 = 5.0 Hz, J_2 = 12 Hz, 8×CH_2), 4.09-3.99 (m, 16H, 16×CH_2), 3.89-3.84 (m, 8H, 8×CH_2), 3.79 (s, 2H, CH_2), 3.65-3.61 (m, 8H, 8×CH), 3.23-3.17 (m, 12H, 6×CH_2), 2.80-2.67 (m, 28H, 14×CH_2), 2.56-2.47 (m, 12H, 6×CH_2), 2.39-2.31 (m, 28H, 14×CH_2), 2.10 (s, 24H, 8×CH_3), 2.05 (s, 24H, 8×CH_3), 2.01 (s, 24H, 8×CH_3), 1.95 (s, 24H, 8×CH_3), 1.88-1.81 (m, 2H, CH_2), 1.24-1.18 (m, 30H, 15×CH_2), 0.84 (t, 3H, J = 6.6 Hz, CH_3); ^{13}C-NMR (100 MHz,

CDCl3) δ 172.77, 170.68, 170.06, 169.74, 145.40, 123.50, 97.56, 69.21, 68.96, 66.31, 65.74, 62.25, 52.89, 52.41, 50.52, 50.40, 50.10, 49.68, 49.14, 45.67, 37.65, 34.73, 33.97, 33.43, 31.98, 30.48, 29.77, 29.58, 29.42, 29.17, 26.68, 22.75, 20.93, 20.79, 14.20, 8.91; HRMS: calcd. for $C_{227}H_{342}N_{48}O_{94}$ $[M+5H]^{5+}$ 1050.2776, found 1050.2773.

Synthesis and characterization of compound II

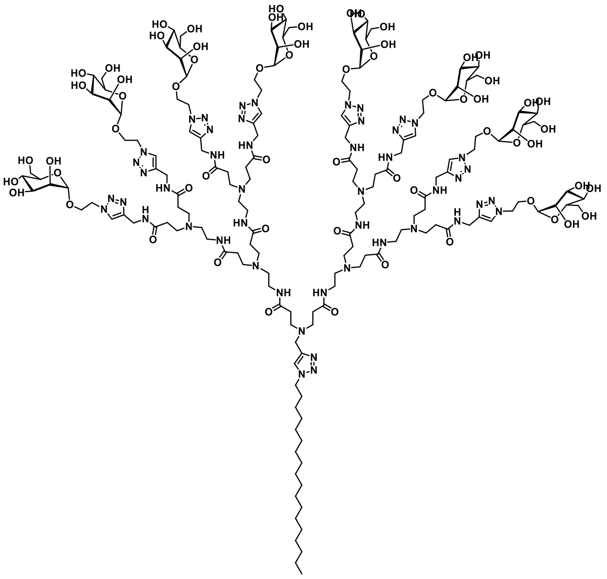

To a solution of compound **13** (110 mg, 0.020 mmol) in MeOH (12 mL) was added aqueous solution of LiOH (31 mg, 1.3 mmol) while stirring under the protection of argon. The reaction mixture was stirred at 25 °C for 3 h. The MeOH was removed by rotavapor and followed by the addition of 1 M HCl solution until the pH = 7. The product was purified by dialysis using dialysis tube of MWCO 2000, followed by lyophilization. After repeating 4 times the operation of dialysis and lyophilization, the product was lyophilized to give compound **II** as a white solid (71 mg, 91%).

^{1}H-NMR (400 MHz, $CD_3OD/CDCl_3$) δ 7.94 (s, 1H, CH), 7.93 (s, 8H, 8×CH), 4.75 (d, 8H, J = 1.7 Hz, 8×CH), 4.64 (t, 16H, J = 5.4 Hz, 8×CH_2), 4.46 (s, 16H, 8×CH_2), 4.41 (t, 2H, J = 7.3 Hz, CH_2), 4.13-4.08 (m, 8H, 8×CH_2), 3.88-3.83 (m, 8H, 8×CH_2, CH_2), 3.80-3.75 (m, 16H, 8×CH_2), 3.67 (dd, J_1 = 5.6 Hz, J_2 = 12 Hz, 8×CH_2), 3.64-3.60 (m, 16H, 16×CH), 3.30-3.25 (m, 12H, 6×CH_2), 3.06-3.02 (m, 8H, 4×CH_2), 2.82-2.75 (m, 28H, 14×CH_2), 2.60-2.59 (m, 12H, 6×CH_2), 2.48-2.40 (m, 28H, 14×CH_2), 1.94-1.91 (m, 2H, CH_2), 1.38-1.30 (m, 30H, 15×CH_2), 0.92 (t, 3H, J = 6.5 Hz,

CH_3); ^{13}C-NMR (100 MHz, $CD_3OD/CDCl_3$) δ 174.20, 174.05, 145.57, 124.94, 100.71, 74.01, 71.94, 71.28, 67.72, 65.97, 62.12, 53.00, 52.21, 51.14, 50.83, 50.53, 37.88, 35.28, 33.83, 33.77, 33.70, 33.40, 32.59, 30.98, 30.26, 30.16, 30.00, 29.74, 27.20, 23.30, 21.52, 14.37; HRMS: calcd. for $C_{163}H_{278}N_{48}O_{62}$ $[M+4H]^{4+}$ 976.5106, found 976.5109.

Cy3/Cy5 encapsulation

Film dispersion method was used to load the Cyanine-3 carboxylic acid (Cy3) (or the Cyanine-5 carboxylic acid (Cy5)) in the amphiphilic dendrimer nanomicelles.[2] Solutions of Cy3 (or Cy5) (1.0 mg/mL) and of dendrimer (1.0 mg/mL) were prepared in methanol. The Cy3 (or Cy5) solution (0.3 mL) was mixed with the dendrimer solution (3.0 mL), then the solvent of methanol was evaporated using vacuum rotary evaporation, and the residue formed a dry film. The dry film was hydrated with water for 5 min under constant stirring. Non-encapsulated dye was separated by filtration through a 0.45-µm polycarbonate membrane (Millipore Co.) and the obtained clear solution was subsequently lyophilized. The amount of encapsulated dye in the dendrimer micelles was measured using florescence spectroscopy. The encapsulation efficiency of dye was calculated around 92 % for both Cy3 and Cy5 as below:

encapsulation efficiency (%): $W_t/W_o \times 100$ %

W_t represents the amount of dye that loaded into nanoparticles; W_o represents the initial amount of dye fed.

NMR spectra of compounds

Figure SA1. (A) ^{1}H-NMR, (B) ^{13}C-NMR and (C) ^{19}F-NMR spectra of dendrimer **3**

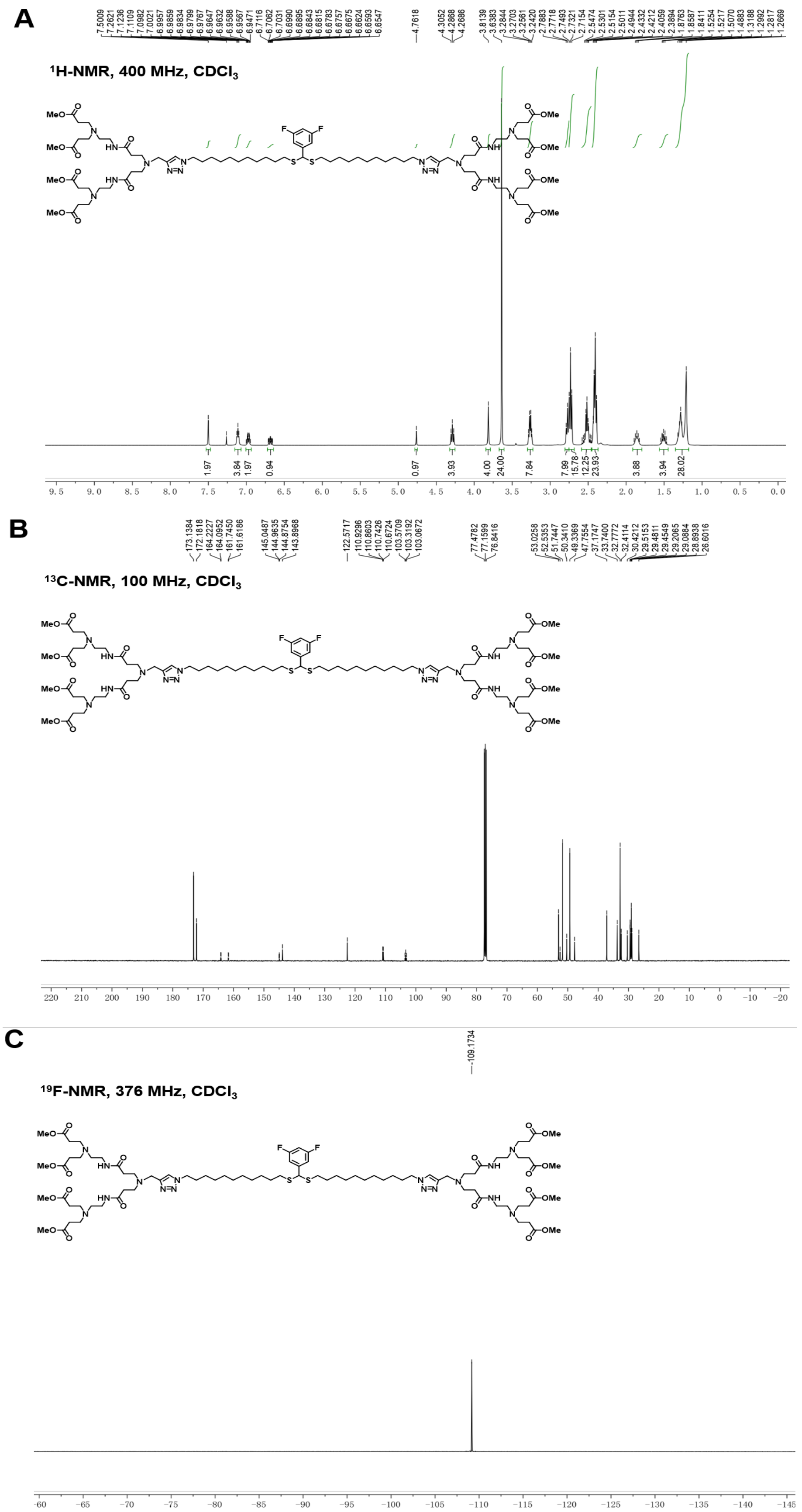

Figure SA2. (A) ^{1}H-NMR, (B) ^{13}C-NMR and (C) ^{19}F-NMR spectra of dendrimer **4**.

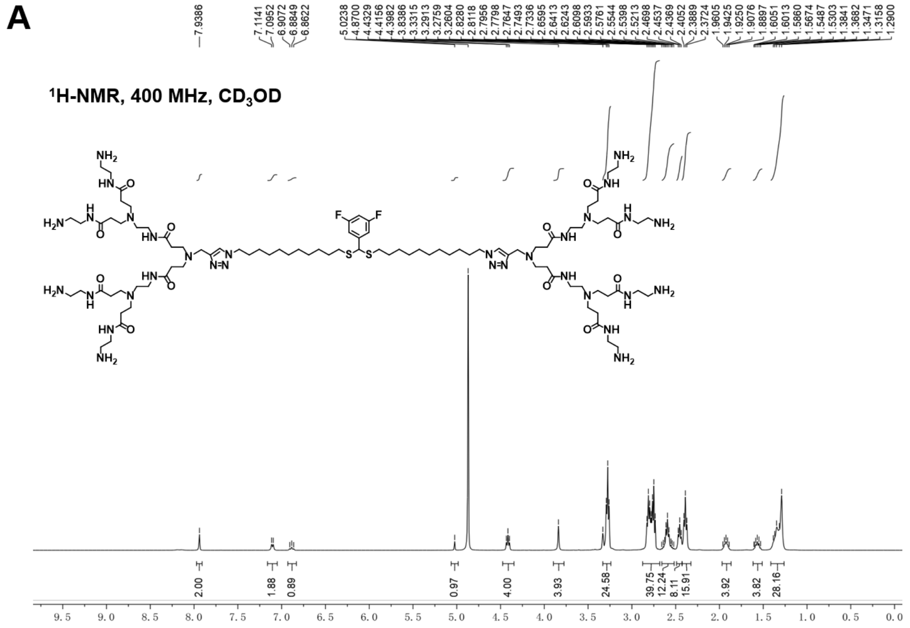

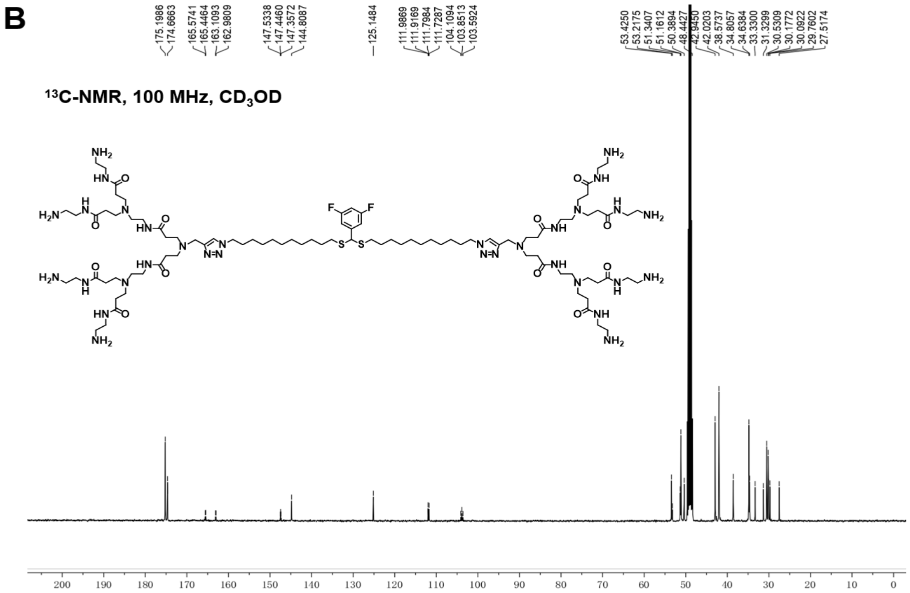

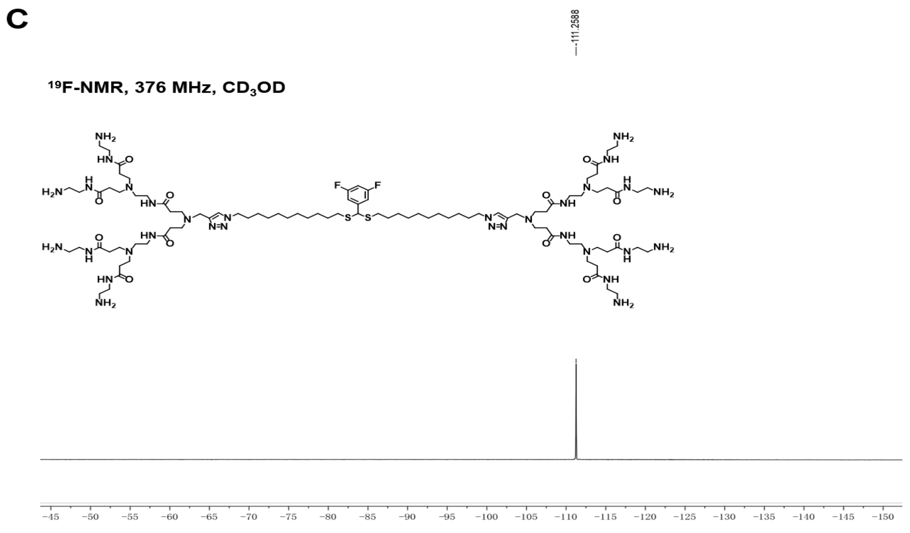

Figure SA3. (A) ^{1}H-NMR, (B) ^{13}C-NMR and (C) ^{19}F-NMR spectra of dendrimer **5**.

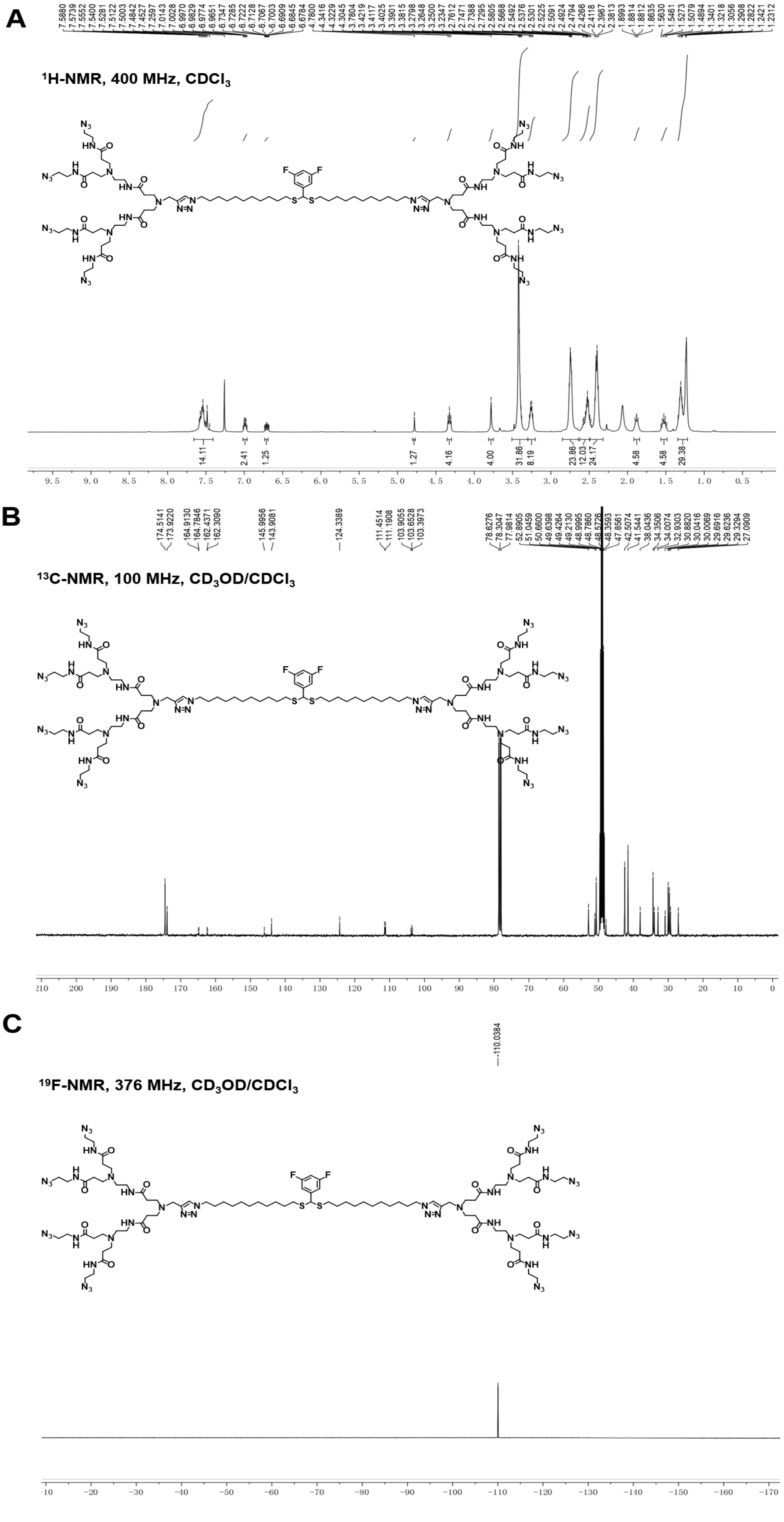

Figure SA4. (A) ^{1}H-NMR, (B) ^{13}C-NMR and (C) ^{19}F-NMR spectra of dendrimer **6**.

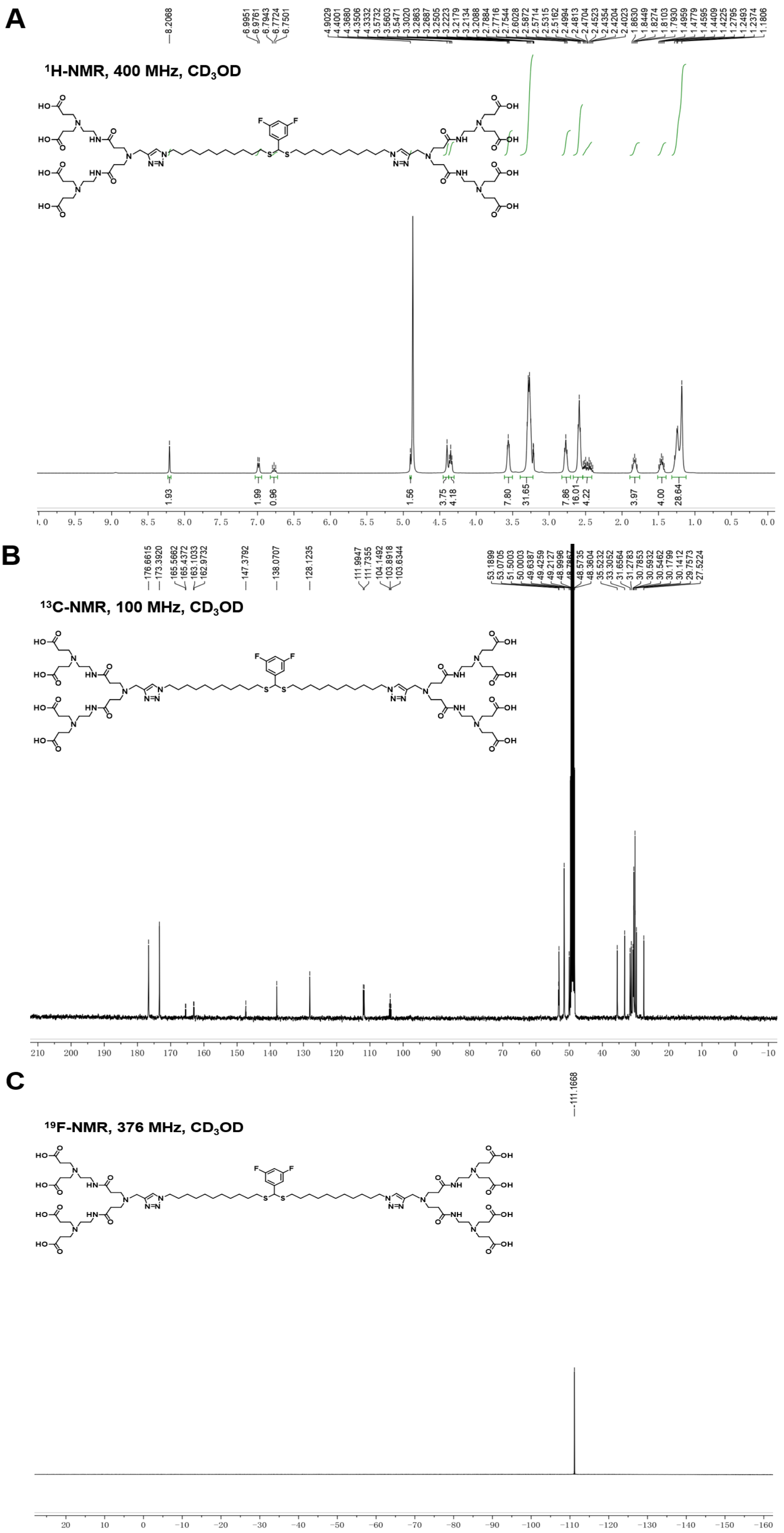

Figure SA5. (A) ^{1}H-NMR, (B) ^{13}C-NMR and (C) ^{19}F-NMR spectra of dendrimer **7**

A

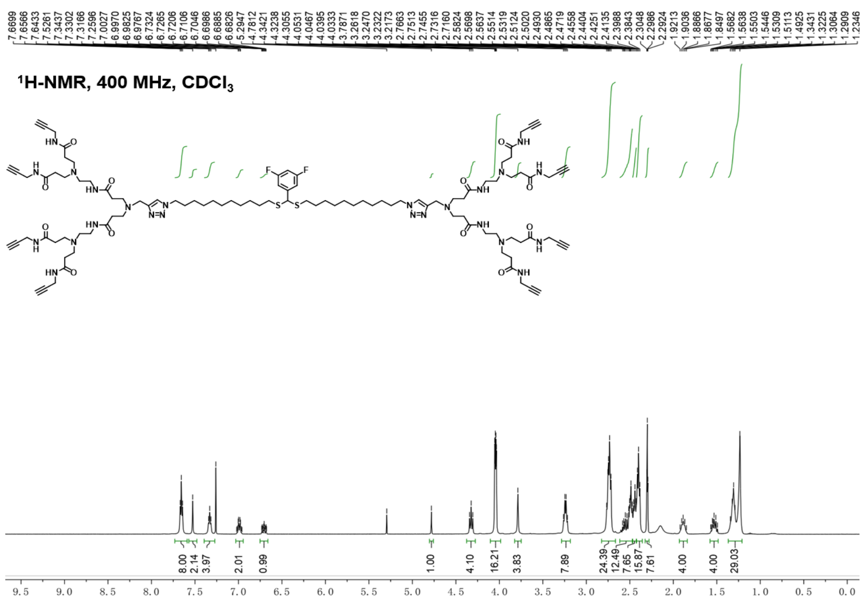

B

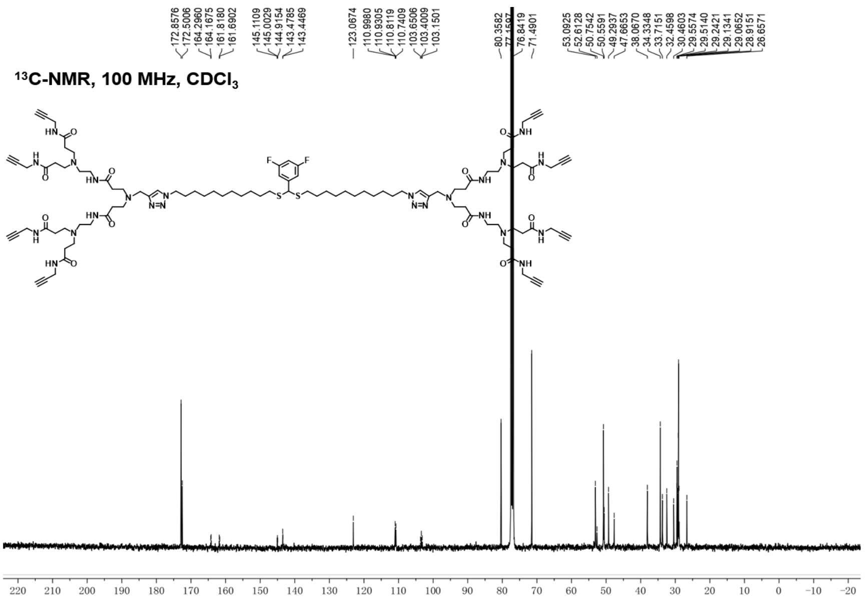

C

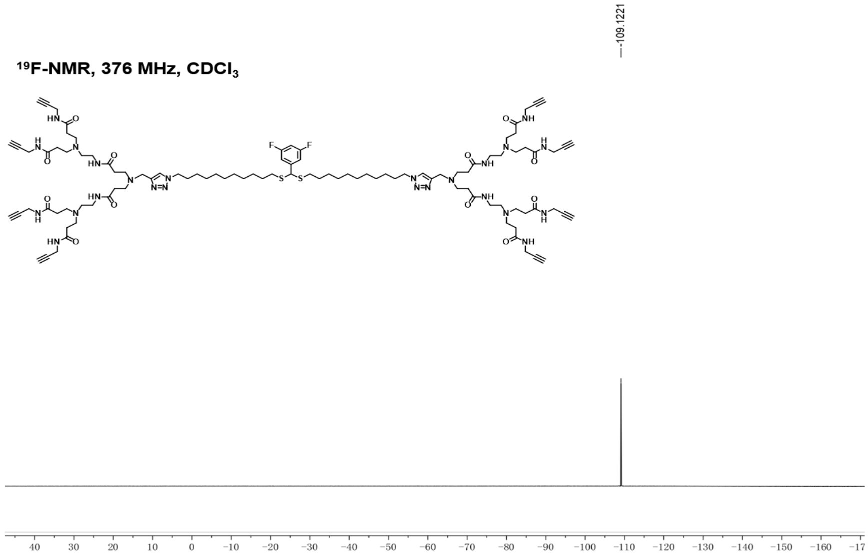

Figure SA6. (A) ^{1}H-NMR, (B) ^{13}C-NMR and (C) ^{19}F-NMR spectra of dendrimer **9a.**

A

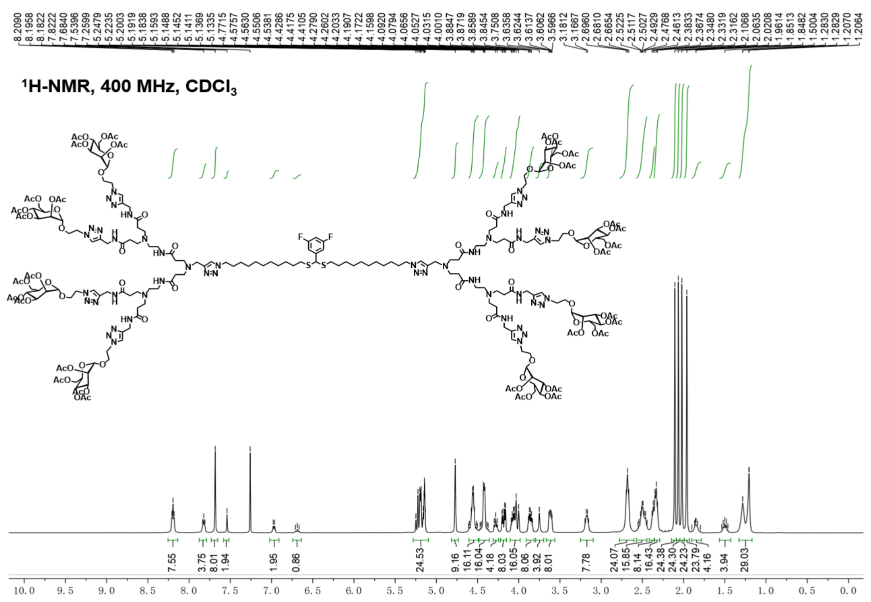

B

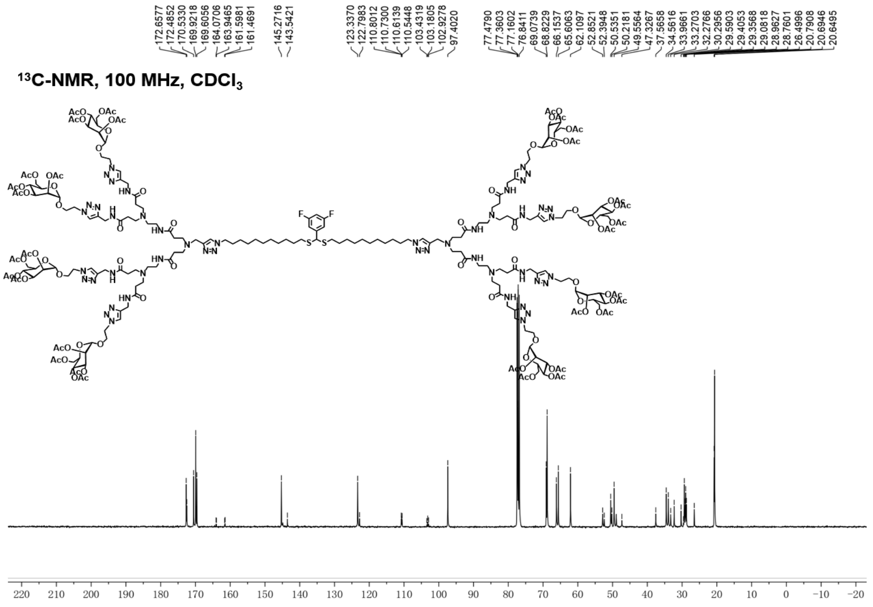

C

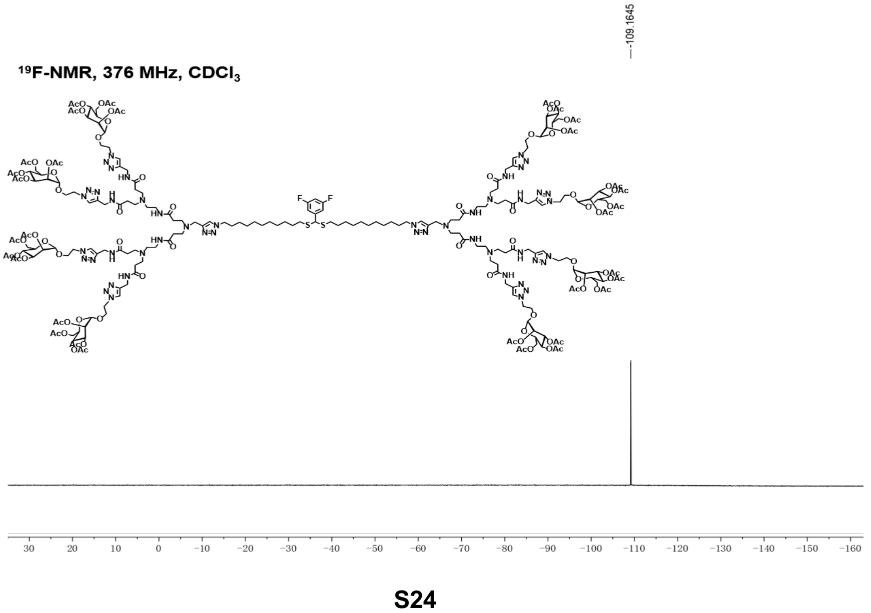

Figure SA7. (A) ^{1}H-NMR, (B) ^{13}C-NMR and (C) ^{19}F-NMR spectra of dendrimer **9b**.

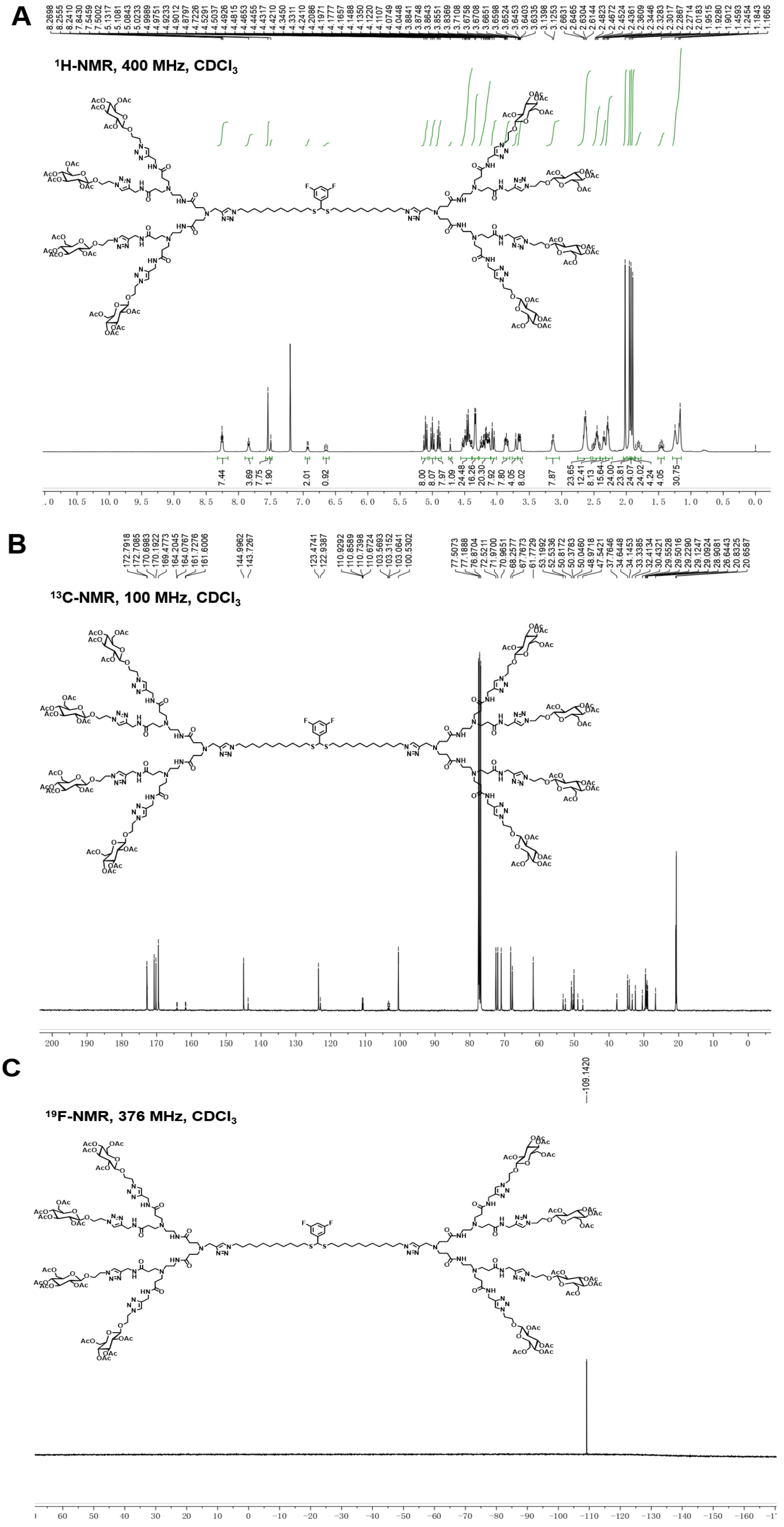

Figure SA8. (A) ^{1}H-NMR, (B) ^{13}C-NMR and (C) ^{19}F-NMR spectra of dendrimer **Ia**.

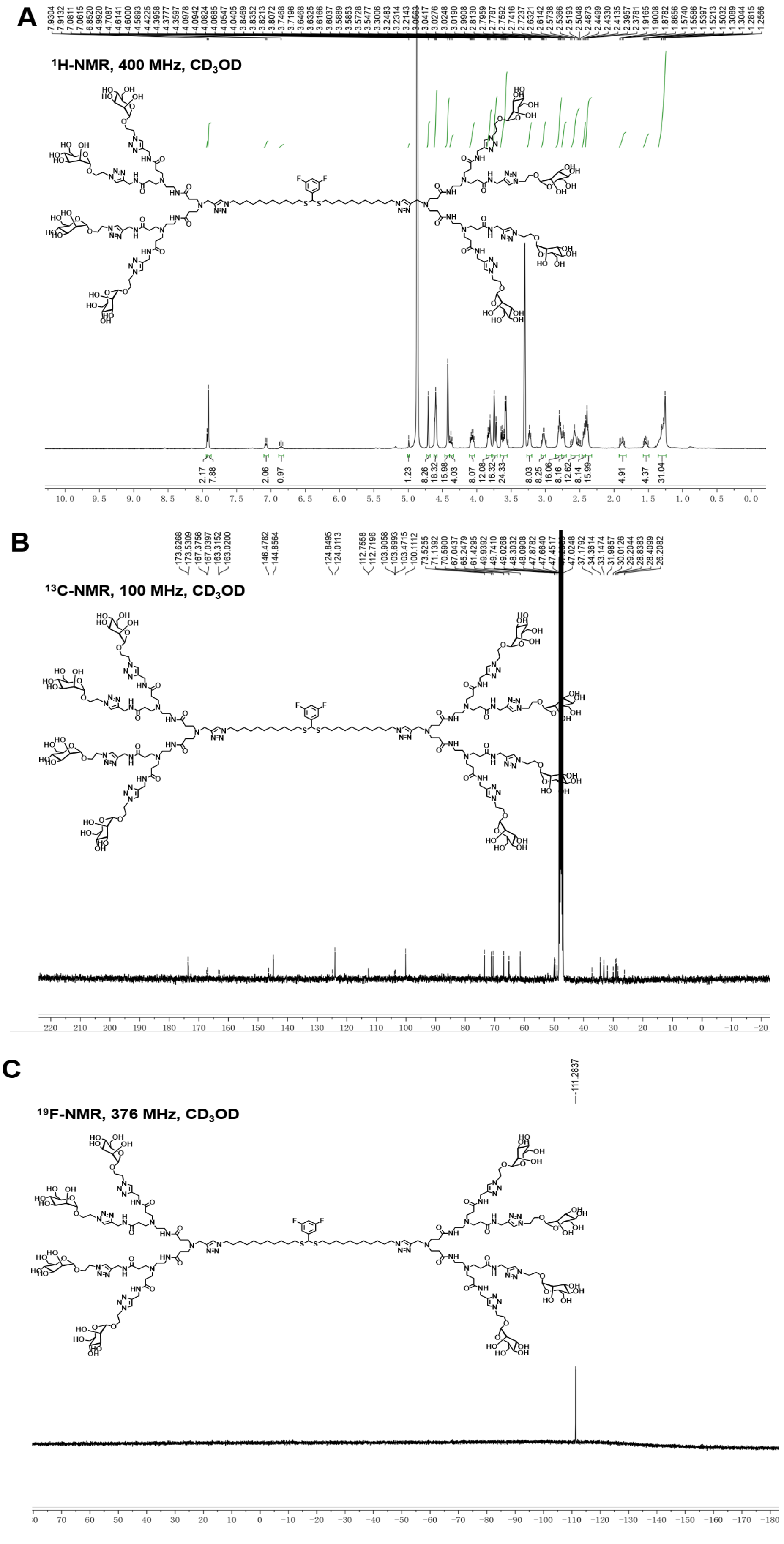

Figure SA9. (A) ^{1}H-NMR, (B) ^{13}C-NMR and (C) ^{19}F-NMR spectra of dendrimer **Ib**.

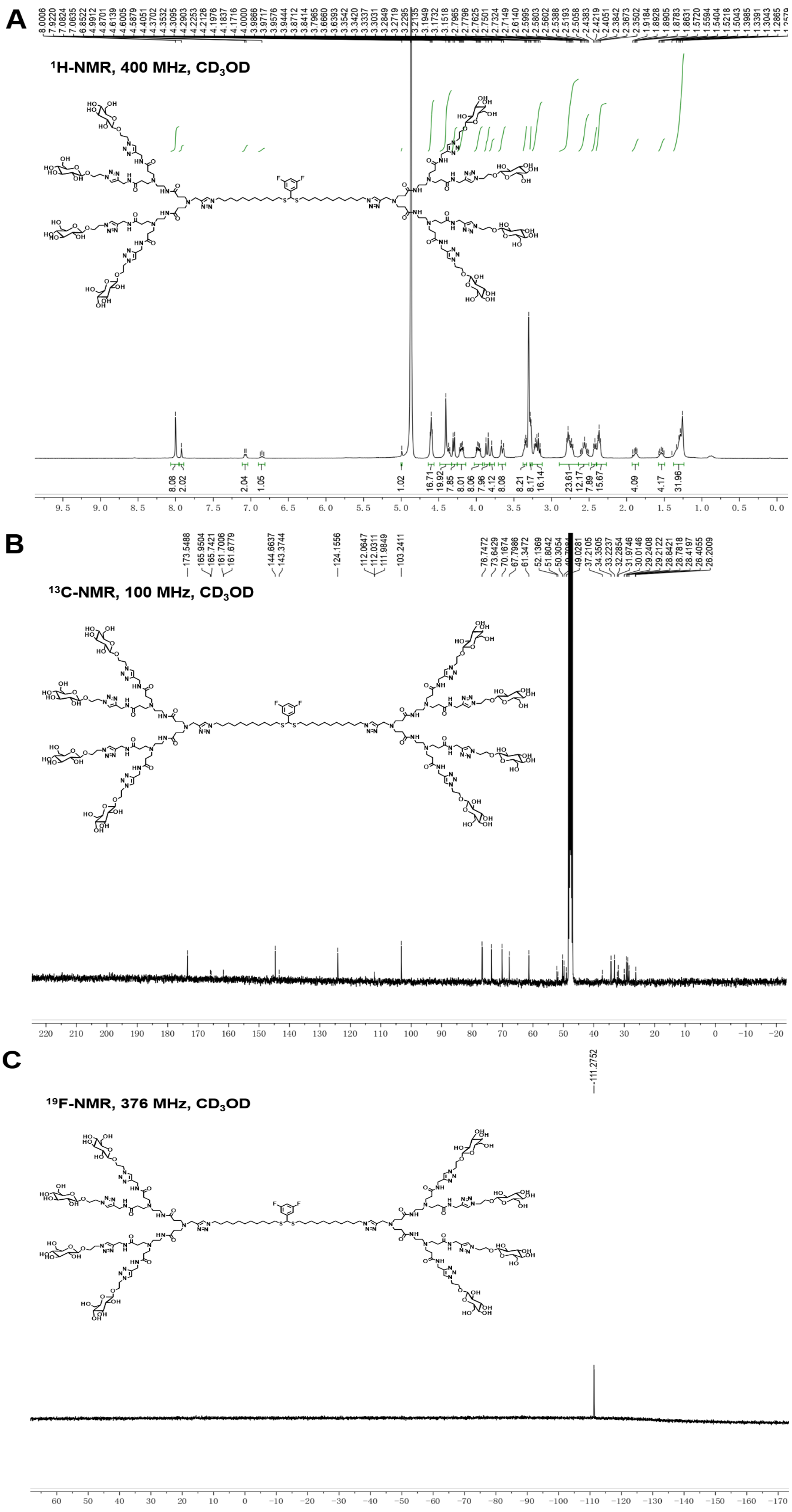

Figure SA10. (A) ^{1}H-NMR and (B) ^{13}C-NMR spectra of dendrimer **11**.

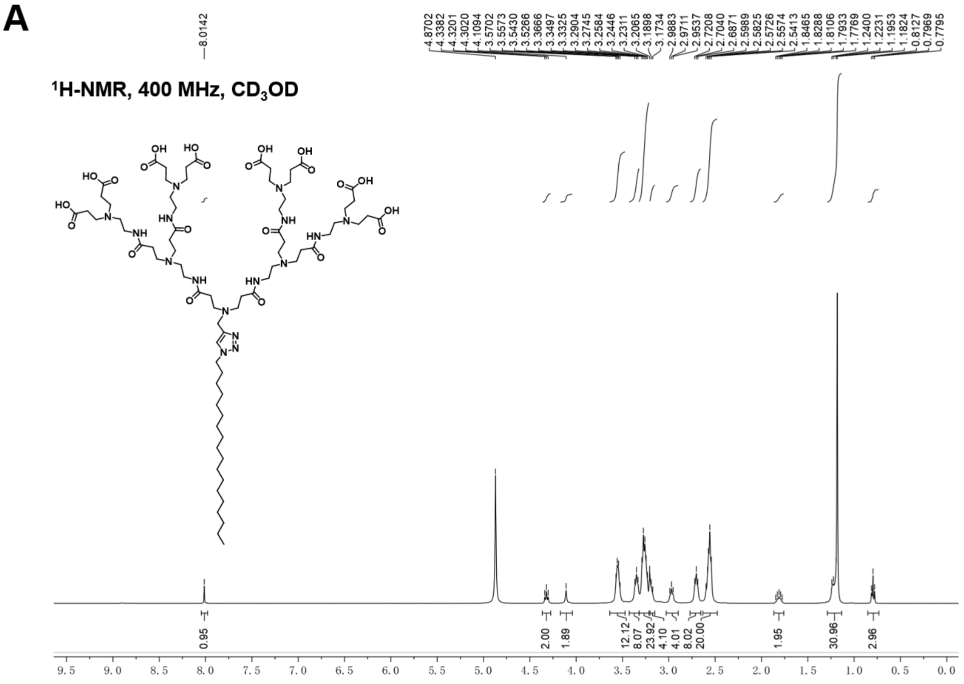

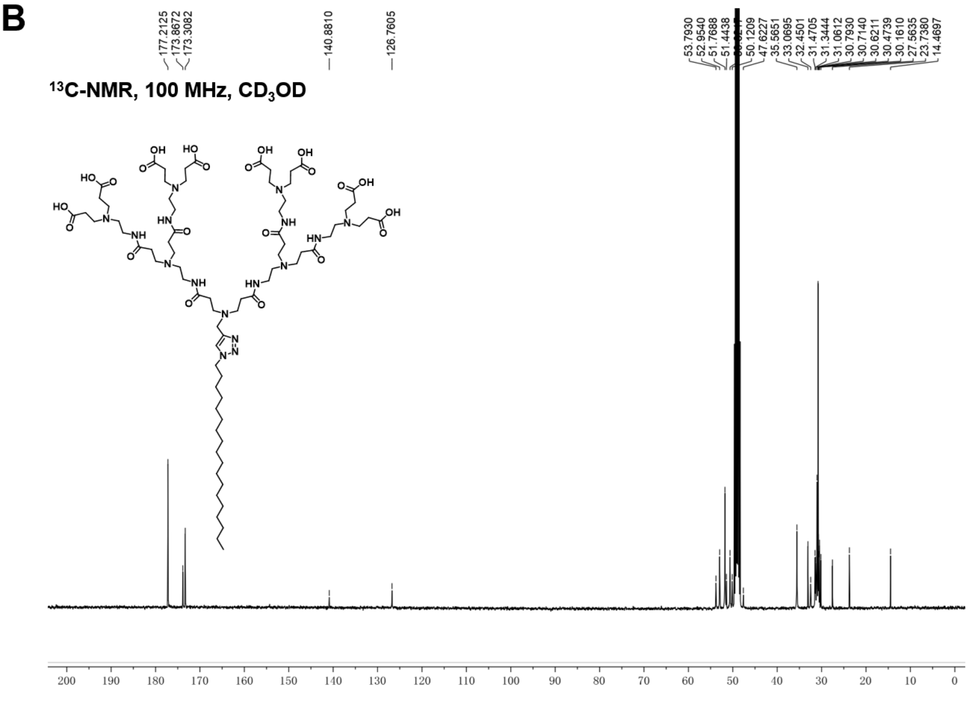

Figure SA11. (A) ^{1}H-NMR and (B) ^{13}C-NMR spectra of dendrimer **12**.

A

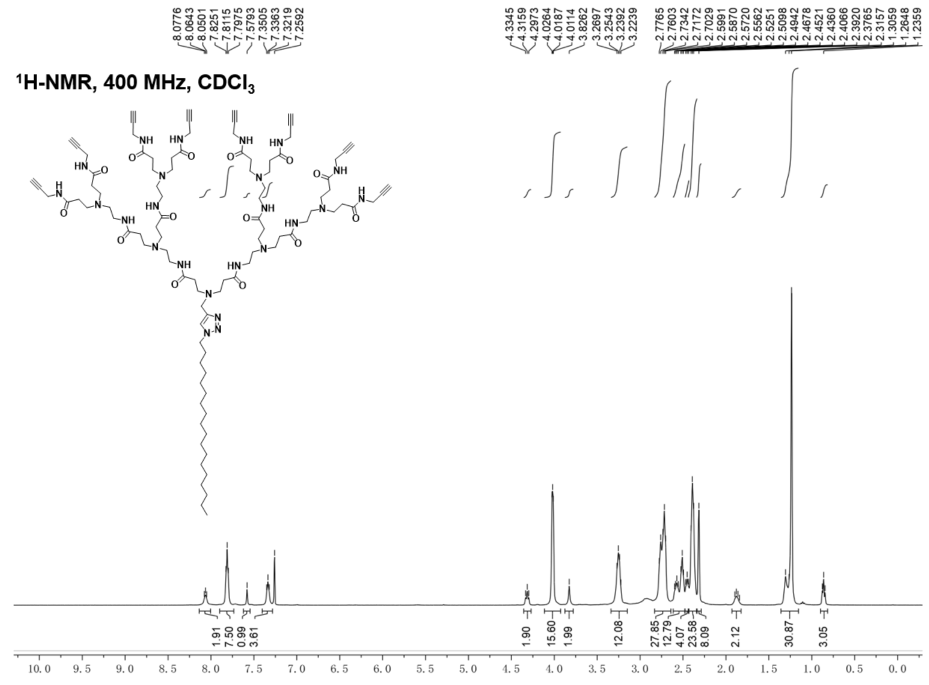

B

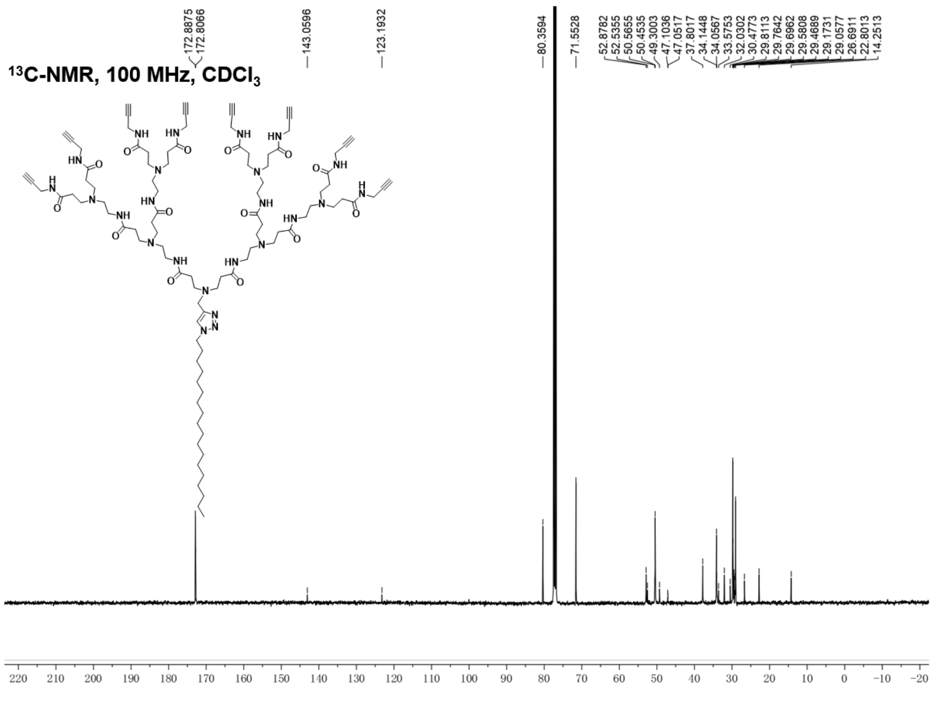

Figure SA12. (A) ^{1}H-NMR and (B) ^{13}C-NMR spectra of dendrimer **13**.

A

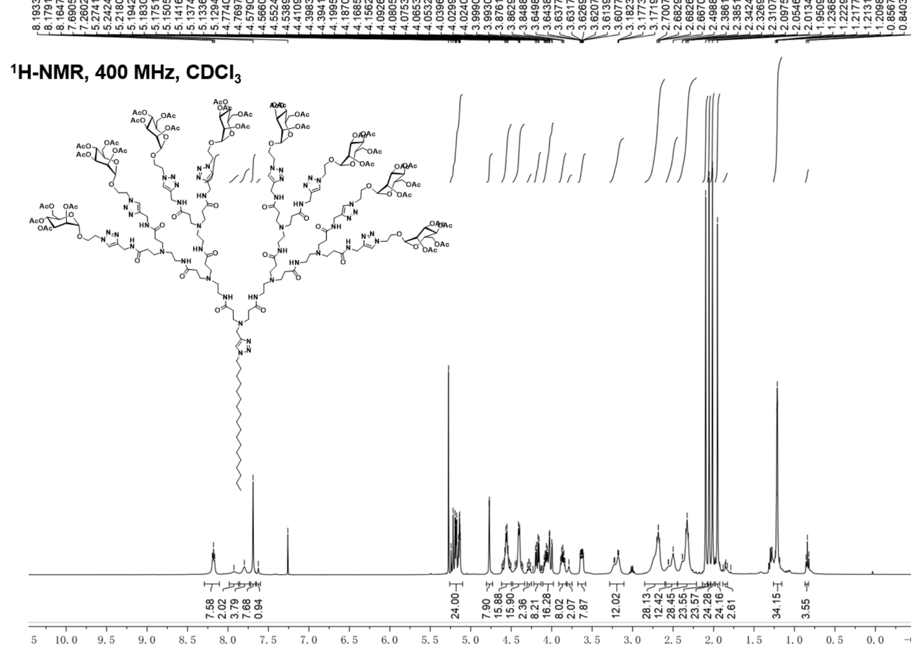

B

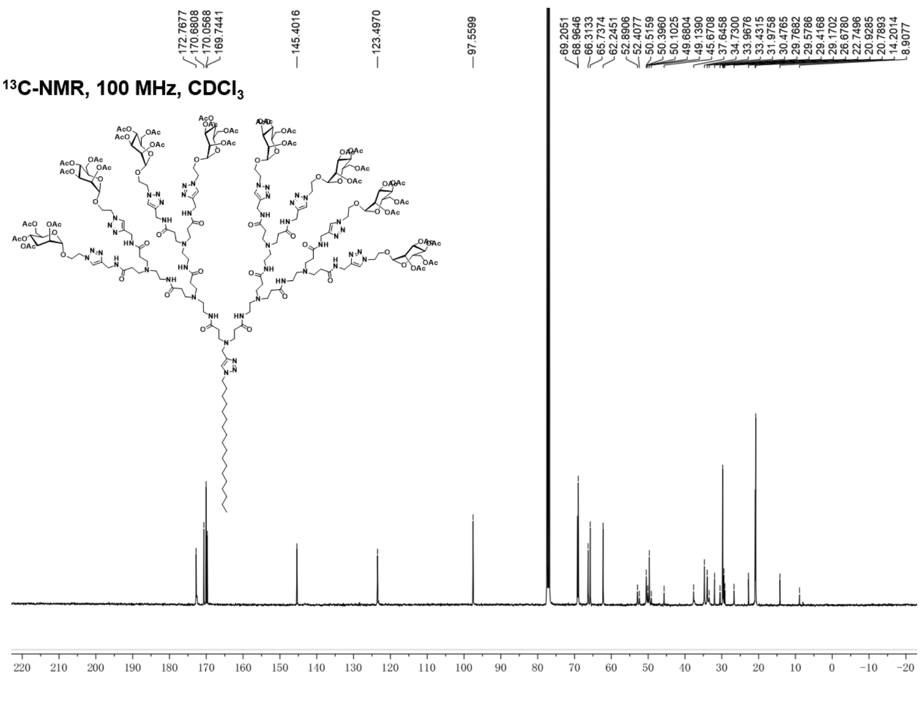

Figure SA13. (A) ^{1}H-NMR and (B) ^{13}C-NMR spectra of dendrimer **II**.

A

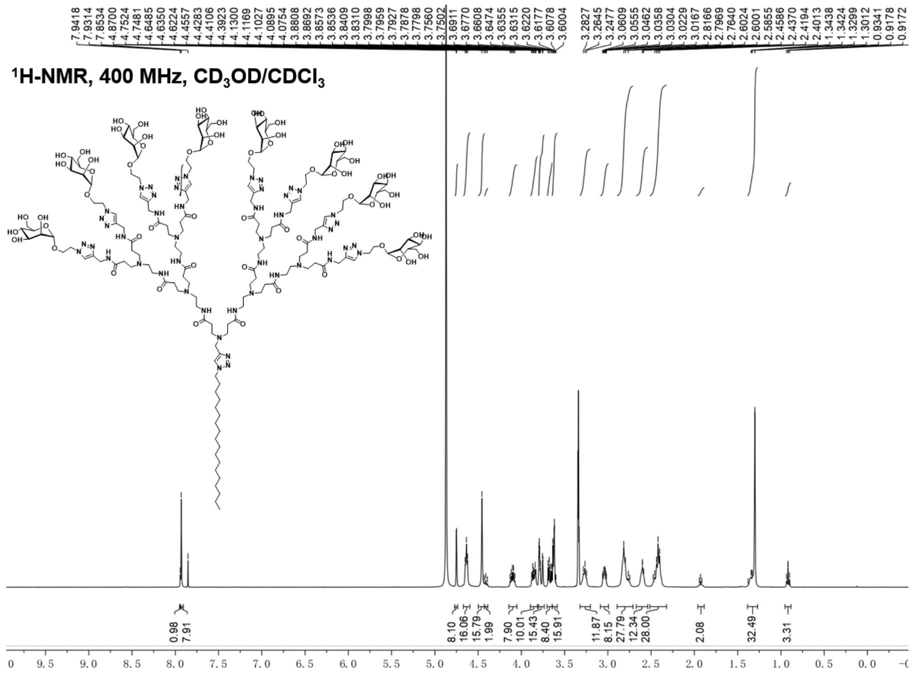

B

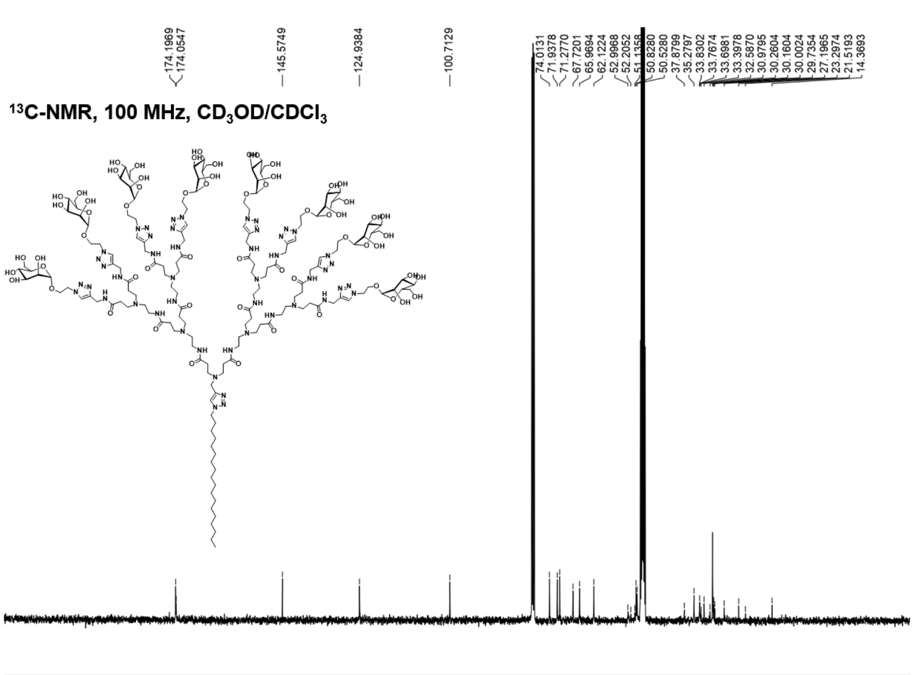

Reference

[1] T. Yu, X. Liu, A. L. Bolcato-Bellemin, Y. Wang, C. Liu, P. Erbacher, F. Qu, P. Rocchi, J. P. Behr, L. Peng, *Angew. Chem. Int. Ed.* **2012**, *51*, 8478-8484.

[2] T. Wei, C. Chen, J. Liu, C. Liu, P. Posocco, X. Liu, Q. Cheng, S. Huo, Z. Liang, M. Fermeglia, S. Pricl, X.-J. Liang, P. Rocchi, L. Peng, *Proc. Natl. Acad. Sci. U. S. A.* **2015**, *112*, 2978-2983.
